# Founder effects and species introductions: A host versus parasite perspective

**DOI:** 10.1111/eva.12868

**Published:** 2019-09-26

**Authors:** April M. H. Blakeslee, Linsey E. Haram, Irit Altman, Kristin Kennedy, Gregory M. Ruiz, A. Whitman Miller

**Affiliations:** ^1^ East Carolina University Greenville NC USA; ^2^ Smithsonian Environmental Research Center Edgewater MD USA; ^3^ University of Southern Maine Portland ME USA

**Keywords:** gene flow, genetic bottleneck, introduction vector, invasion, life cycle, propagule pressure, trematode

## Abstract

Species colonizations (both natural and anthropogenic) can be associated with genetic founder effects, where founding populations demonstrate significant genetic bottlenecks compared to native populations. Yet, many successfully established free‐living species exhibit little reduction in genetic diversity—possibly due to multiple founding events and/or high propagule pressure during introductions. Less clear, however, is whether parasites may show differential signatures to their free‐living  hosts. Parasites with indirect life cycles may particularly be more prone to founder effects (i.e., more genetically depauperate) because of inherently smaller founding populations and complex life cycles. We investigated this question in native (east coast) and introduced (west coast) North American populations of a host snail *Tritia obsoleta* (formerly *Ilyanassa obsoleta*, the eastern mudsnail) and four trematode parasite species that obligately infect it. We examined genetic diversity, gene flow, and population structure using two molecular markers (mitochondrial and nuclear) for the host and the parasites. In the host snail, we found little to no evidence of genetic founder effects, while the trematode parasites showed significantly lower genetic diversity in the introduced versus native ranges. Moreover, the parasite's final host influenced infection prevalence and genetic diversity: Trematode species that utilized fish as final hosts demonstrated lower parasite diversity and heightened founder effects in the introduced range than those trematodes using birds as final hosts. In addition, inter‐regional gene flow was strongest for comparisons that included the putative historical source region (mid‐Atlantic populations of the US east coast). Overall, our results broaden understanding of the role that colonization events (including recent anthropogenic introductions) have on genetic diversity in non‐native organisms by also evaluating less studied groups like parasites.

## 1 INTRODUCTION

Founder effects are a commonly recognized genetic signature of newly established populations following successful colonization events (Barton & Charlesworth, 1984). During these events, just a subset of a species’ source genetic diversity may be carried to a novel location, resulting in substantially lower genetic diversity compared to the source (Barton & Charlesworth, 1984; Grosberg & Cunningham, 2001; Holland, 2000). Examples can be found across taxa, region, and habitat type, including human colonization of landmasses out of Africa (Ramachandran et al., 2005); native and non‐native colonization by Eurasian plants (Eckert, Manicacci, & Barrett, 1996); and non‐native colonization of the Caribbean by the Indo‐Pacific lionfish (Betancur et al., 2011). The severity and maintenance of reduced genetic diversity associated with founder events will depend on multiple factors, notably the size of the founding population (i.e., founding genetic diversity and impact of genetic drift and selection), the level of isolation of the founding population and number of colonization events (i.e., extent of gene flow), and the length of time following the initial migration event (i.e., new diversity accrued through mutations and gene flow) (Austerlitz, Jung‐Muller, Godelle, & Gouyon, 1997; Baker & Jenkins, 1987; Barton & Charlesworth, 1984; Carlton, 1996; Hufbauer, Rutschmann, Serrate, Conchard, & Facon, 2013). Many of these factors also influence the likelihood of species colonization and are typically referred to as “propagule” or “colonization” pressure (e.g., Holle & Simberloff, 2005; Lockwood, Cassey, & Blackburn, 2005; Lockwood, Cassey, & Blackburn, 2009; Miller & Ruiz, 2009; Ricciardi, Jones, Kestrup, & Ward, 2010).

**Figure 1:**
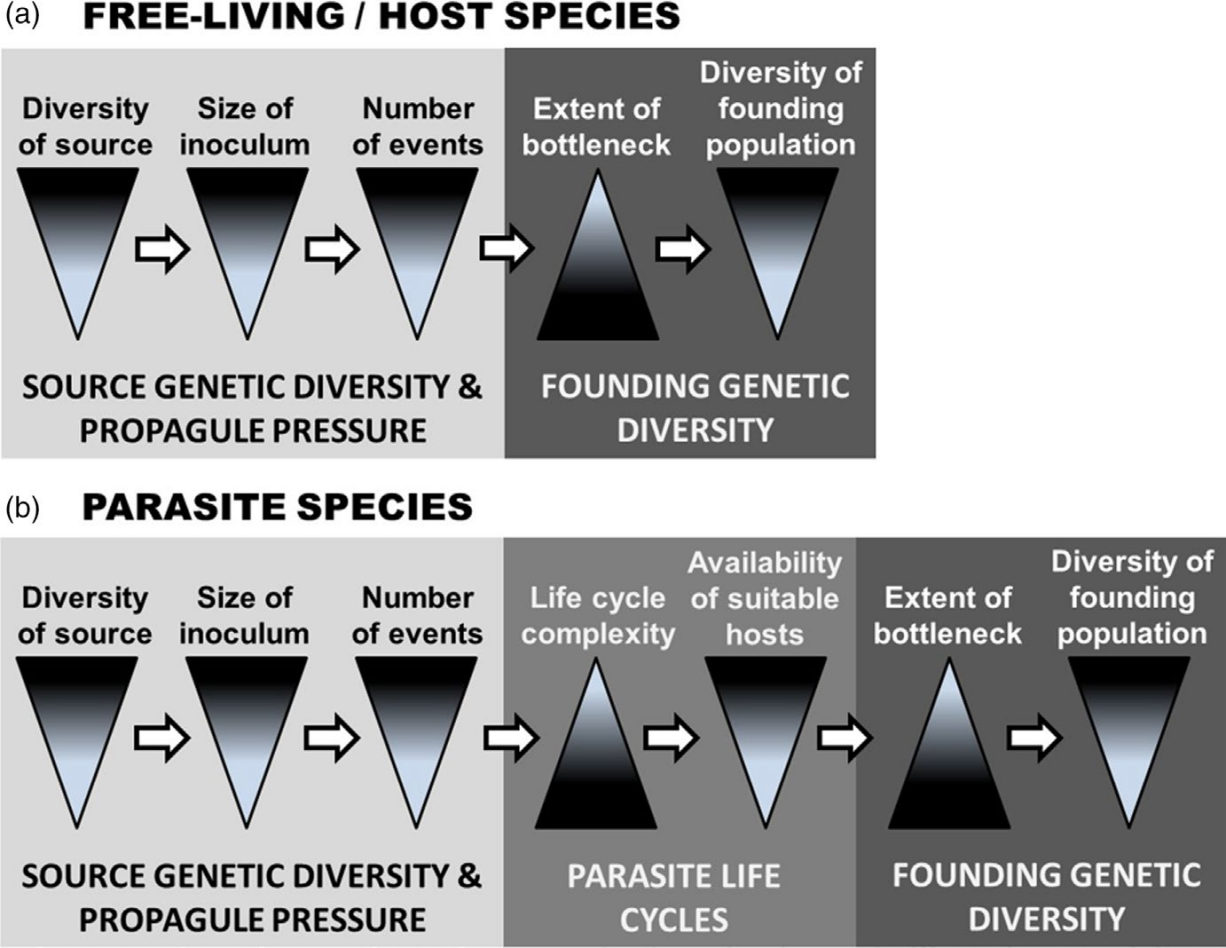
Theoretical schematic for the differences in (a) hosts and (b) parasites with indirect life cycles that may lead to differential genetic diversity in their founding regions. For (a) hosts, source genetic diversity and propagule pressure will affect the extent of the genetic bottleneck in a founding region, and therefore the genetic diversity in the region. For (b) parasites with indirect life cycles, life cycle complexity (e.g., multi‐host trophically transmitted parasites) and availability of suitable hosts will additionally affect bottlenecks and founding diversity. Each triangle depicts the directional change in genetic diversity (width) with increase (from top to bottom) in the individual factors labeled at top. Parasite life cycles include additional factors that modify diversity in founding populations. This figure has been adapted from figure 1 in Roman and Darling (2007) and figure 7.2 in Blakeslee (2016) with permission from the authors

Although the establishment of founding populations can result from natural or anthropogenic events (Fontdevila, 1989), many recent species colonizations are due to human‐mediated transfer mechanisms (“species introduction vectors”), which have accelerated with human globalization in the past century (Ruiz, Fofonoff, Carlton, Wonham, & Hines, 2000). A wide diversity of introduction vectors are responsible for carrying biota to new, historically inaccessible regions and may be intentional (e.g., food, biological control, ornamental use) or unintentional (e.g., species associated with vessels, bait, live oysters) (Carlton, 1996, 2003; Cohen & Carlton, 1998; Fowler et al., 2016; Ruiz, Carlton, Grosholz, & Hines, 1997; Seebens, Schwartz, Schupp, & Blasius, 2016; Williams et al., 2013). In addition, vectors may operate only once (acute) or over a period of years (chronic), possibly supporting the introduction of new species and more individuals of already established species to novel regions (Azmi, Hewitt, & Campbell, 2014; Carlton & Geller, 1993; Minchin, Gollasch, Cohen, Hewitt, & Olenin, 2009; Ruiz et al., 1997). While founder effect signatures are common in many species introductions (e.g., Betancur et al., 2011; Blakeslee, Byers, & Lesser, 2008; Planes & Lecaillon, 1998), numerous examples also exist of introduced populations retaining high levels of genetic diversity with little or no genetic bottleneck. Roman and Darling (2007) suggested that this “genetic paradox” was likely due to the inherent complexity and particularities of each introduction event, including their size and frequency (i.e., propagule pressure), their timing, their effective population size, and their vector type (Darling, Bagley, Roman, Tepolt, & Geller, 2008; Geller, Darling, & Carlton, 2010; Roman & Darling, 2007; Voisin, Engel, & Viard, 2005). For example, Roman (2006) found a strong “diluting [of] the founder effect” in non‐native populations of European green crab (*Carcinus maenas*) resulting from multiple introduction events originating from different regions in the native range. More specifically, those introduction vectors with higher entrained propagule pressure can enable transfer of greater genetic variability to founding populations, lessening the depression of genetic diversity (Figure 1). Moreover, multiple introduction events can lead to genetic admixture of varied genotypes across a species’ source range, possibly promoting successful colonization and secondary spread (Lehnert et al., 2018; Wagner, Ochocki, Crawford, Compagnoni, & Miller, 2017).

For human‐mediated introductions, most of the research on genetic founder effects has focused on free‐living species, and much less is known about these effects in parasites (e.g., parasites were not included in the review by Roman & Darling, 2007). A couple notable exceptions where genetic diversity in native and non‐native populations has been co‐investigated in hosts and parasites include *Battilaria attramentaria* (Asian horn snail), which invaded the US west coast with cryptic lineages of trematode parasites (Miura, Torchin, Kuris, Hechinger, & Chiba, 2006), and *Littorina littorea* (common periwinkle snail), where host diversity and parasite diversity were used to help resolve its cryptogenic status in eastern North America (Blakeslee et al., 2008). In both studies, parasites were integral to the understanding of host–parasite dispersal mechanisms and sources. Generally, however, parasites are excluded from the majority of population and community‐level surveys and investigations, despite being extremely important members of aquatic and terrestrial communities (Kuris et al., 2008; Rohde, 2002; Thompson, Mouritsen, & Poulin, 2005). Parasites are also integral to the evolutionary and ecological trajectories of their hosts across region and system (Choisy, Brown, Lafferty, & Thomas, 2003; Lafferty, 1999; Lafferty & Kuris, 2009; Mouritsen & Poulin, 2002) and are increasingly being recognized as important contributors to ecosystem health, restoration, biodiversity, and invasion history (Altman & Byers, 2014; Blakeslee et al., 2008; Byers, Altman, Grosse, Huspeni, & Maerz, 2011; Byers, Blakeslee, Linder, Cooper, & Maguire, 2008; Criscione, Cooper, & Blouin, 2006; Hechinger, Lafferty, Huspeni, Brooks, & Kuris, 2007; Hudson, Dobson, & Lafferty, 2006; Huspeni, Hechinger, & Lafferty, 2005; Huspeni & Lafferty, 2004; Mackenzie, 1999; Vidal‐Martínez & Wunderlich, 2017). Specific to the latter, although hosts and parasites may show many congruent signatures during anthropogenic introduction events (e.g., Blakeslee et al., 2008), they may demonstrate disparate patterns in terms of genetic founder effects. This is because parasites typically have more limited chances for invasion than hosts, since only a subset of invading hosts will be infected upon introduction. In addition, complex life cycles could make reproductive success more challenging, especially for parasites requiring transmission through multiple and often species‐specific hosts (Shoop, 1988). As a result, smaller parasite founding populations would be more subject to evolutionary forces like drift that tend to reduce genetic variability. Further, if founding individuals cannot find mates and successfully reproduce, genetic diversity will be further reduced in newly formed parasite populations (Chang, Blakeslee, Miller, & Ruiz, 2011; Johannesson, 2016; Taylor & Hastings, 2005). Indeed, these so‐called Allee effects could be heightened by a parasite's complex life cycle (Deredec & Courchamp, 2006), which requires the parasite to find and infect appropriate hosts vital to life cycle completion (Figure 1).

**Figure 2:**
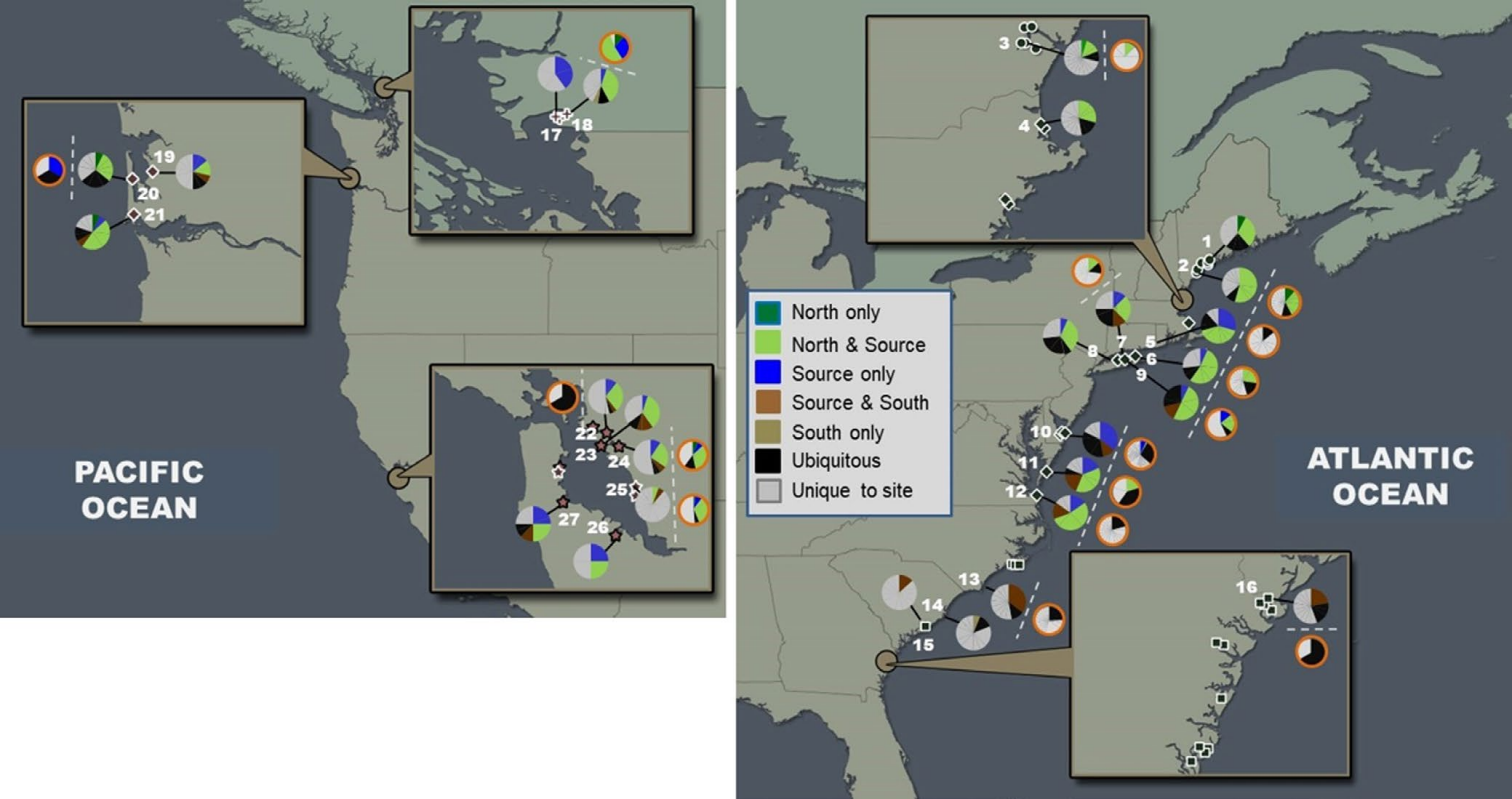
Sample locations in the introduced Pacific and native Atlantic regions of *Tritia obsoleta* and four of its trematode parasites. In the native region, small black circles represent the *North* subregion; black diamonds represent the Source subregion; and black squares represent the *South* subregion. In the introduced region, red crosses represent Boundary Bay (*BB)*, red diamonds represent Willapa Bay (*WB)*, and red stars represent San Francisco Bay (*SFB)*. Numbers represent those sites included in genetic analyses (sites are identified in Appendix S1B); all other sites on the map were sampled for parasite prevalence and richness only (see Blakeslee et al., 2012). Haplotype (COI) frequencies for *T. obsoleta* and trematode parasites are portrayed as pie charts, with pie piece coloring defined in the Key. Trematode pie charts are distinguished by an orange border. In some sites, we were unable to pair *T. obsoleta* and trematode haplotype data due to small sample size for the trematodes (i.e., sites where prevalence of infection was low). In the key, “*North* only,” “Source only,” and “South only” refer to haplotypes found at a site that were only found in a particular subregion; “*North* & *Source”* and “*Source* & South” refer to haplotypes in sites that were found in both the *North* and *Source* subregions, or the *Source* & *South* subregions (note: there were no haplotypes found at any site that were shared between just the *North* & *South* subregions); “Ubiquitous” represents haplotypes found across all three subregions; and “Unique to site” refers to haplotypes only found in a particular site. Figure modified from Blakeslee et al. (2012) with the authors’ permissions

Here, we compared native and non‐native genetic diversity associated with host and parasite populations in a system where an introduced host snail (*Tritia obsoleta*, formerly *Ilyanassa obsoleta*) is infected by several species of trematode parasites in both its native region of eastern North America and its non‐native region of western North America. This study system allowed us to test for differences in multiple parasite species with different life cycles while controlling for the host species. The host and its parasites also reflect a relatively recent founding event, making our study particularly well suited for examining questions about the effects of introduction events on genetic diversity. Based on our expectations for differential signatures in the host versus parasites, we tested the following hypotheses: (1) *T. obsoleta's* trematode parasites would show a stronger genetic bottleneck than the snail host in introduced versus native regions and subregions, due to inherent differences in parasite and host propagule pressure and life cycles (Figure 1); (2) final hosts would have a strong influence on genetic diversity of trematodes in native versus introduced regions, given potential differences in final host dispersal and availability in these regions; (3) the highest levels of gene flow between the regions would originate from the putative source subregion (Mid‐Atlantic), for both the host and parasites. We examined genetic diversity across a broad geographic range in the native versus introduced regions for host and parasites using two population genetic markers: the cytochrome oxidase I (COI) mitochondrial marker and the 18S rRNA nuclear marker.

## 2 STUDY SYSTEM

### 2.1 North American distribution and invasion history of *Tritia obsoleta*

*Tritia obsoleta* (Family Nassariidae) is an estuarine snail (up to ~30 mm shell length) with an extensive eastern North American native range, occurring from the Gulf of Saint Lawrence, Canada, to northern Florida, USA (Abbott, 1974). It typically lives in brackish, soft‐sediment habitats (e.g., salt marsh, mudflats) often at high densities (>1,000/m^2^) (Cranford, 1988; Appendix S1A). The snail has broad temperature and salinity tolerances (0–30°C; 10–35 PSU) and is omnivorous, feeding on detritus, carrion, and plants/algae; it lays egg capsules on structures such as algae, grass blades, and shell, and its veliger larvae hatch as free‐floating plankton before settling along the shore (Fofonoff, Ruiz, Steves, Hines, & Carlton, 2018). The snail was likely introduced to the North American Pacific coast via large‐scale intentional but ultimately failed attempts at transplanting the eastern oyster, *Crassostrea virginica*, to the west coast in the 1900s (Carlton, 1992). Its first Pacific records were in San Francisco Bay (*SFB*), California, in 1907 (Carlton, 1992); Willapa Bay (*WB*), Washington, in 1945; and Boundary Bay (*BB*), British Columbia, in 1952 (Demond, 1952). Both historical evidence and ecological evidence suggest that the introduction originated from Long Island Sound and nearby Mid‐Atlantic bays and estuaries. Oysters were targeted from these areas because they were hardier and more tolerant of long‐distance travel (Kochiss, 1974; Miller, 2000).

### 2.2 Trematode life cycles and parasite distributions

For this investigation, we focused on digenean trematodes: flatworm parasites that utilize multiple hosts to complete life cycles (Rohde, 2005). Trematodes typically use gastropods as first‐intermediate hosts, where they reproduce asexually, proliferating in the gonad and ultimately castrating the snail. During this phase, a free‐swimming, infective stage called cercaria is produced. Cercariae emerge from the snail and, depending on the species of trematode, seek out a wide range of second‐intermediate hosts, such as mollusks, worms, crustaceans, and fish. Once a suitable host is located, the parasite may encyst as a metacercaria within the second‐intermediate host. The parasite is trophically transmitted to a final vertebrate host (often shorebird or fish) when the intermediate host is ingested by the final host. Inside the final host's gut, the parasite sexually reproduces. Parasite eggs are transmitted to the environment through the final host's feces. The snail then contracts infection passively or actively: Passive transmission occurs when the snail accidentally ingests the parasite eggs, which hatch within the snail as the miracidial stage; and active infection occurs when the parasite miracidia hatch from the eggs in the environment and actively infect the snail (Combes, Fournier, Mone, & Theron, 1994; Rohde, 2005).

There are nine documented trematode species that infect *T. obsoleta* in its native range, and they utilize an array of downstream hosts (Figure S1; Blakeslee et al., 2012; Curtis, 1997, 2007; Phelan, Blakeslee, Krause, & Williams, 2016). Most (7/9) infect three hosts, and all but one use either fish (*n* = 4) or shorebirds (*n* = 4) as final hosts. Five of *T. obsoleta's* trematodes have been discovered in the introduced range: all five in *SFB* (Blakeslee et al., 2012; Grodhaus & Keh, 1958) and two in *WB* and *BB* (Blakeslee et al., 2012; Table 1). Trematode prevalence is variable across sites and subregions, with a notable pattern associated with the type of final hosts: Trematodes using fish as final hosts (hereafter “fish‐using”) are five times less common in the introduced versus native ranges, while for those trematodes using birds as final host (hereafter “bird‐using”), prevalence is similar between the ranges (Blakeslee et al., 2012). Prior demographic surveys have documented differences in native and introduced trematode diversity in *T. obsoleta;* yet little is known about how they may differ genetically. One prior genetic study using allozymes found limited genetic structure in native *T. obsoleta* populations (Gooch, Smith, Knupp, 1972). Two more recent studies include *T. obsoleta* as part of multi‐host, multi‐parasite examinations of correlative patterns between parasite escape and genetic bottlenecks in native and introduced regions (Blakeslee & Fowler, 2012; Blakeslee, 2016). These studies found varying support for heightened bottlenecks in parasites versus hosts across several intermediate hosts (Blakeslee, 2016; Blakeslee & Fowler, 2012). For *T. obsoleta's* trematodes, some recent barcoding research has linked upstream and downstream hosts (Phelan et al., 2016). To date, however, there is no detailed, comparative genetic study of the snail host and its trematode parasites in the two ranges.

**Table 1:** Prevalence of trematode infection in *Tritia obsoleta's* native and introduced regions in eastern and western North America, respectively

Region or subregion	Sites	Snails dissected	Snails infected	Total sp. richness	Abs prev (all trematodes) (%)	Avg prev (all trematodes) (%)	*SE* prev (all trematodes) (%)	*AV* avg prev (%)	*HQ* avg prev (%)	*LS* avg prev (%)	*ZL* avg prev (%)	Bird‐using (%)	Fish‐using (%)
Source	18	2,660	720	8	27.07	24.76	4.53	0.55	2.40	5.56	3.50	1.48	4.53
North	12	2,047	807	8	39.42	37.96	8.65	0.63	4.68	5.31	7.08	2.66	6.20
South	19	1,955	327	8	16.73	16.74	3.43	0.05	0.24	5.81	0.54	0.15	3.17
SFB	8	1,364	134	5	9.82	8.44	4.75	0.91	4.74	1.03	0.36	2.82	0.70
WB	5	669	18	2	2.69	2.93	2.32	3.07	0.73	0.00	0.00	1.90	0.00
BB	5	540	22	2	4.07	3.80	2.62	0.64	2.20	0.00	0.00	1.42	0.00
Native	49	6,662	1854	9	27.83	24.88	3.16	0.41	2.44	5.56	3.71	1.43	4.63
Introduced	18	2,573	174	5	6.76	5.62	2.30	1.54	2.56	0.34	0.12	2.05	0.23

Note

The native region is divided into subregions based on the source area for the introduction (*Source*), north of the source (*North*), and south of the source (*South*). The introduced region is divided into subregions based on bays where the snail is introduced, including San Francisco Bay (*SFB*) in California, Willapa Bay (*WB*) in southern Washington, and Boundary Bay (BB) in northern Washington/ southern British Columbia. The first column denotes the region or subregion; the second column is the number of sites sampled per subregion/region; the third column includes the total number of snails dissected for trematodes per subregion/region; the fourth column is the total number of infected snails per subregion/region; the fifth column is the total species richness of trematodes detected in the subregion/region; the sixth column is the absolute prevalence (abs prev) of all trematodes [# infected/ # dissected] per subregion/region; the seventh column depicts the average prevalence (avg prev) of all trematodes within a subregion/region; the eighth column is the prevalence standard error (*SE*) of all trematodes within a subregion/region; the ninth through twelfth columns depict the average prevalence of the four trematodes analyzed in this study (*AV = Austrobilharzia variglandis; HQ = Himasthla quissitensis; LS = Lepocreadium setiferoides; ZL = Zoogonus lasius*) within a subregion/region; the thirteenth and fourteenth columns are the average prevalence of trematodes using birds or fish as final hosts.

## 3 METHODS

### 3.1 Trematode prevalence and richness sampling

From 2009 to 2012, *T. obsoleta* were collected from 49 native sites (*n* = 6,662 snails) from Maine to Georgia (Table 1; Figure 2). Using historical evidence, *T. obsoleta* populations were divided into three subregions based on the putative source area and sites to the north and south. The *Source* subregion had 18 sites (*n* = 2,660 snails); the *North* had 12 sites (*n* = 2,047 snails); and the *South* had 19 sites (*n* = 1,955 snails). In the introduced region, *T. obsoleta* was grouped by its three discrete bays: *SFB*: eight sites (*n* = 1,364 snails), *WB*: five sites (*n* = 669 snails), and *BB*: five sites (*n* = 540 snails). Adult snails were collected haphazardly by hand in the low intertidal zone at daily low tide by walking parallel to the water's edge. Approximately 100 snails were collected per site and dissected in the laboratory using standard protocols (Blakeslee et al., 2012) to measure trematode richness and prevalence per site. Trematodes were identified to species level using published keys and images (Curtis, 1997, 2007) and later confirmed with genetic analysis (see below).

### 3.2 Genetic diversity sampling

A subset of sites, snails, and trematodes were processed for genetic analyses (Figure 2). For *T. obsoleta*, the following were the sample sizes for COI DNA sequencing—*Source*: *n* = 8 sites, *n* = 127 individuals; *North*: *n* = 4 sites, *n* = 75 individuals; *South*: *n* = 4 sites, *n* = 66 individuals; *SFB*: *n* = 6 sites, *n* = 106 individuals; *WB*: *n* = 3 sites, *n* = 43 individuals; and BB: *n* = 2 sites, *n* = 24 individuals. A subset of these were also sequenced using the 18S marker (native: *n* = 39; introduced: *n* = 30). Early processing revealed little variation among individuals and populations using the 18S marker; thus, no subregional examinations were performed for this marker. For trematodes, we included the four most prevalent species in the two regions: *Austrobilharzia variglandis* (*AV*), *Himasthla quissitensis* (*HQ*), *Lepocreadium setiferoides* (*LS*), and *Zoogonus lasius* (*ZL*). The following were our trematode sample sizes for the COI marker: *Source*: *n* = 8 sites, *n* = 163 individuals; *North*: *n* = 4 sites, *n* = 43 individuals; *South*: *n* = 2 sites, *n* = 32 individuals; *SFB*: *n* = 4 sites, *n* = 58 individuals; *WB*: *n* = 1 site, *n* = 3 individuals; and BB: *n* = 2 sites, *n* = 18 individuals. Trematodes were inherently more difficult to detect in the introduced region, leading to unequal sample sizes; as a result, some subregional comparisons were limited. 18S was used as a second marker for the trematode (*HQ*) with the most equivalent sample sizes between the regions. In some analyses, trematodes were grouped by final host taxa: *AV* and *HQ* are bird‐using and *LS* and *ZL* are fish‐using (Blakeslee et al., 2012; see Appendices S1B–S1E for site details of host and parasites).

### 3.3 DNA sequencing, alignment, and phylogenetic analyses

DNA was extracted from the snail's foot using a standard cetyl trimethyl ammonium bromide (CTAB) protocol (France, Rosel, Agenbroad, Mullineaux, & Kocher, 1996). For trematodes, multiple cercariae and rediae/sporocysts were extracted from the gonad of infected snails, and DNA was extracted using the same CTAB method. Most of these individuals should represent genetic clones as a result of asexual reproduction, though it is possible that multiple infections of the same or different trematode species could occur within a single snail host, but this would be rare. COI and 18S primers and fragment sizes for the snail and trematodes can be found in Table S1. PCR was performed using the following PCR profile: 95°C for 2 min; 30 cycles of 95°C for 30 s, 55°C for 30 s, and 72°C for 60 s; and 72°C for 5 min (Steinberg, Krimsky, & Epifanio, 2008). For all samples, sequencing was performed in both the forward and reverse directions at the Smithsonian Institution's Laboratories of Analytical Biology (Washington, DC, USA). Sequences were assembled and manually inspected for ambiguities using Geneious 10.1.2 (Biomatters Ltd). Sequences were aligned without gaps using the ClustalW algorithm (Larkin et al., 2007) and collapsed into haplotypes using TCS v.1.21 (Clement, Posada, & Crandall, 2000). The optimal nucleotide substitution model was selected based on the Akaike information criterion in jModelTest in Geneious 10.1.2 (Darriba, Taboada, Doallo, & Posada, 2012). The selected model was then used in Bayesian phylogeny reconstructions using MrBayes (Huelsenbeck & Ronquist, 2001) in Geneious 10.1.2 (see Table S1 for information on phylogenetic rooting). Rarefaction curves were constructed using EstimateS 8.20 (Colwell et al., 2012) to estimate haplotype diversity in each population and to quantify the effects of sampling effort on resulting haplotype diversity in the snail host and its four trematodes. GenBank accession numbers for the host snail and its trematodes are as follows: MN272433–MN272597 and MN272598– MN272732 (Blakeslee et al., 2019).

### 3.4 Genetic diversity, population structure, and migration rates

For both the snail and trematodes, we calculated fixation indices for population pairs based on pairwise differences between haplotypes (*φ*_ST_), and tested significance of differentiation in Arlequin. Hierarchical analysis of molecular variance (AMOVA) was used to estimate variation for *T. obsoleta* between regions; between native subregions and the introduced region; between subregions across the two regions; and between subregions within regions (see Table S2 for all comparisons). Because 18S demonstrated little genetic variation for *T. obsoleta*, just the regional comparison was made for this marker. Similarly for *HQ*, we performed a subset of comparisons. Haplotypes were visualized in PopArt (Leigh & Bryant, 2015) using a TCS haplotype network for both the host and its parasites. In addition, pairwise *φ*_ST_ results were visualized with a nonmetric multidimensional scaling (nMDS) analysis (using Primer 6; Plymouth Marine Laboratory) to look for spatial patterns between and among populations. The spatially closest populations are most genetically similar and could reveal likely source populations. Using the same regional and subregional comparisons presented in Table S2, we also examined gene flow using IMa (Hey & Nielsen, 2007). IMa is a coalescent‐based program that uses Markov chain Monte Carlo (MCMC) sampling and applies the isolation with migration model to estimate migration rates (m1/*μ* and m2/*μ*, where *μ* is the mutation rate per site) between two populations assumed to have shared a common ancestor. We performed 10 replicate runs of each comparison, which included 30 chains of at least two million steps per chain after an initial burn‐in period of 100,000 steps; we ended runs when posterior density parameter curves were stable (Hey, 2009).

## 4 RESULTS

### 4.1 Founder effects in host versus parasites

**Figure 3:**
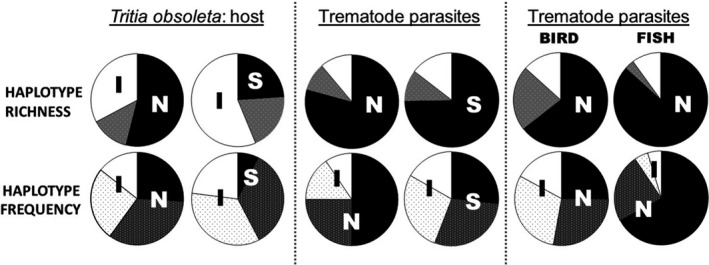
Pie chart representations of proportional haplotype richness and frequency based on the COI marker for the host, *Tritia obsoleta*, and four of its trematode parasites (*Austrobilharzia variglandis*, *Himasthla quissitensis*, *Lepocreadium setiferoides*, *Zoogonus lasius*). Three separate comparisons are made for proportional haplotype richness and frequency: left—*T. obsoleta*, the host snail; middle—all four trematode parasites collectively; right—trematodes divided into bird‐using (*AV* and *HQ*) and fish‐using (*LS* and *ZL*) groups. For both the left and middle panels, the left pie charts compare the introduced versus entire native region, and the right compares the introduced region to just the source area of native region; the far right panel compares the whole native region to the introduced region for different definitive hosts. Black pie pieces = haplotypes only found in the native (N) or Source (S) regions; white pie pieces = haplotypes only found in the introduced (I) region; gray patterned pieces = haplotypes shared between the regions. For haplotype frequencies: black‐patterned pieces = occurrences of shared haplotypes across native and introduced that are found in the native or Source regions (shared‐native); white‐patterned pieces = occurrences of shared haplotypes that are found in the introduced region (shared‐introduced)

To determine whether genetic founder effects differed between the host and parasites in the native versus introduced regions (Hypothesis 1), we examined genetic diversity using two markers (COI and 18S). In *T. obsoleta*, we detected 165 COI haplotypes across the two regions: 89 (54%) were only in the native region, 54 (33%) only in the introduced region, and 22 (13%) shared between regions. Haplotype richness was marginally higher (*Χ* ^2^ = 3.313, *p* = .069) in the native versus introduced region. Interestingly, however, the opposite was found between the putative *Source* and the introduced region, the latter having significantly higher haplotype richness (*Χ* ^2^ = 4.2733, *p* = .039; Figure 3). When examining haplotype frequencies, shared haplotypes were very common between the native and introduced regions (60% of all occurrences), and this increased to 70% for comparisons with the *Source*. Connectivity was high between regions and subregions. Among the shared haplotypes, there were few sequence changes (Figure S2), and haplotypes were broadly represented among the subregions. Moreover, 55% of haplotypes in the introduced region originated from the *Source* and *North* subregions. In analyses using the 18S marker, we found limited genetic variation among *T. obsoleta* individuals and populations. Just five haplotypes were detected, the majority (91%) being a single, dominant one that was ubiquitous across sites in both regions (Appendix S1C). Altogether, three haplotypes were detected in the native region and two in the introduced.

**Figure 4:**
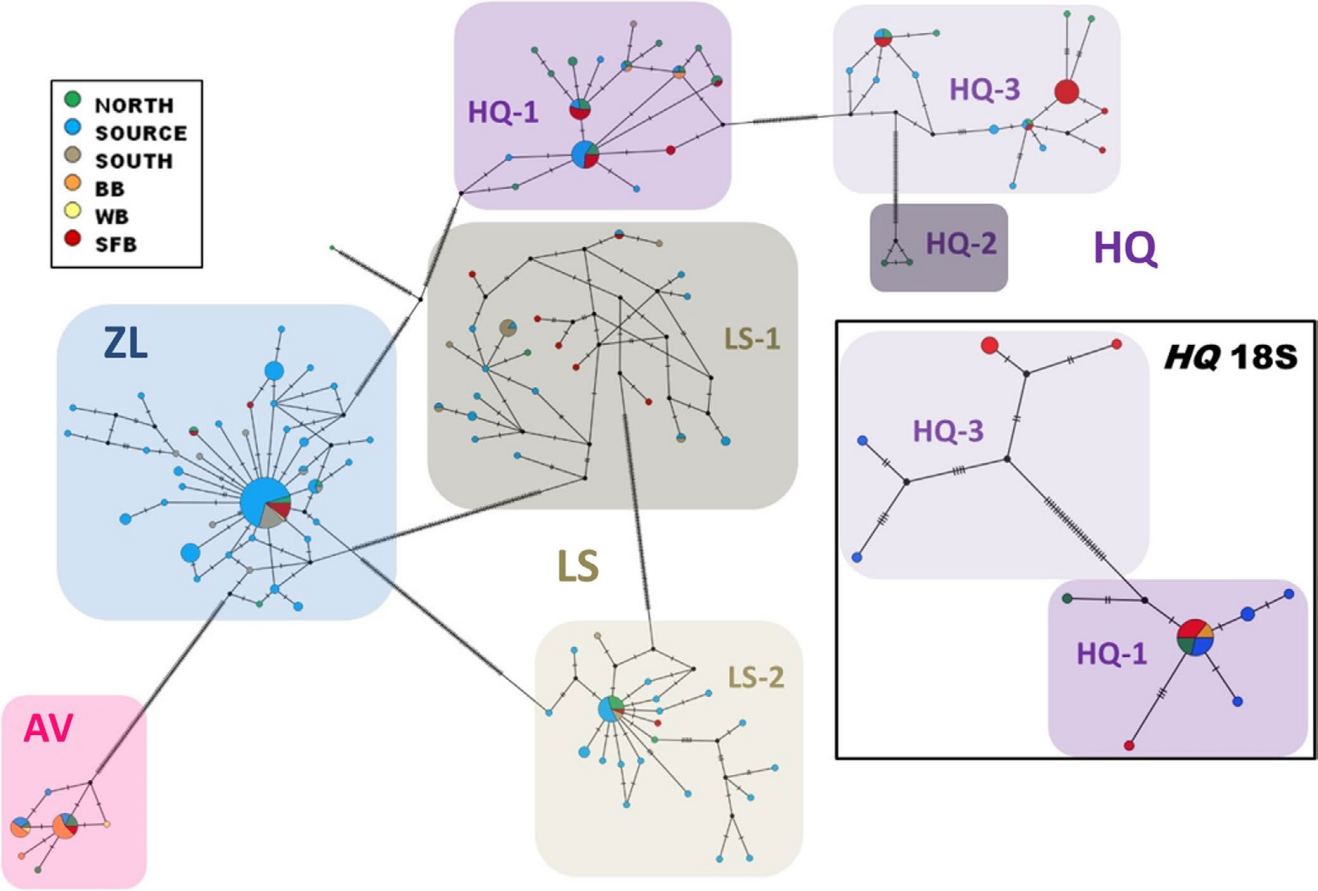
Haplotype network depicting COI maker of the four trematode parasites (*Austrobilharzia variglandis *[*AV*], *Himasthla quissitensis *[*HQ*], *Lepocreadium setiferoides *[*LS*], *Zoogonus lasius *[*ZL*]) of *Tritia obsoleta* in the native and introduced regions, and 18S marker of *HQ*. Colors represent different subregions in the native and introduced regions. Shading depicts the different trematode species and genetically distinct lineages (HQ‐1, HQ‐2, HQ‐3, LS‐1, and LS‐2) based on a Bayesian phylogenetic tree (Figure S3). The inset represents the analysis for *HQ* using the 18S marker. See Table 1 for subregion abbreviations and trematode species abbreviations

For the trematodes, the COI marker revealed a large degree of genetic diversity (*n* = 135 haplotypes; Appendix S1D), but this was largely attributed to native richness, with a total of 107 haplotypes (79%) only in the native range, 15 (11%) only in the introduced, and 13 (10%) shared between regions. Haplotype richness was significantly (Χ^2^ = 31.650, *p* < .00001) greater in the native versus introduced region and also the *Source* versus introduced region (Χ^2^ = 18.206, *p* = .00002) (Figure 3). Similar patterns were found for haplotype frequencies, where the majority of occurrences were in the native region or shared between regions. Similar to the snail host, geographic analyses found strong connections between the introduced region and the *Source* and *North* subregions (Figure 2). In addition, both the haplotype network and phylogenetic tree (Figures 4 and S3) revealed clear separation into distinct lineages based on trematode species. Notably, two trematode species were revealed to have genetically distinct lineages within their species complexes: *HQ* with three and *LS* with two lineages. In *HQ*, two of these lineages were also found using the 18S marker.

In rarefaction analyses, only a fraction of the host snail's predicted genetic diversity was captured in the two ranges—likely due to the number of singleton haplotypes found in both regions and the number of unshared haplotypes detected in the introduced region (Figure S4; Appendix S1B). Haplotype accumulation curves suggested thousands of *T. obsoleta* sequences would be necessary to reach an asymptote in genetic diversity, which was predicted to be much greater in the native versus introduced region. In contrast, though the expected *Source* richness was closer to the introduced richness, the latter was actually predicted to attain higher diversity. In trematode analyses, predicted diversity in the native region was much greater than observed, but the asymptote in predicted introduced richness was more similar to what we actually observed. Extrapolation curves demonstrated substantially higher (8×) native versus introduced richness, while the predicted *Source* richness was about 2–3× higher.

### 4.2 Life cycle influences

To test how the intricacies of trematode life cycles may differentially affect diversity in the two regions (Hypothesis 2), we examined trematode prevalence in host snails and also genetic diversity in trematode species with different final hosts (fish or birds). Trematode infection prevalence was 4× greater in the native versus introduced regions for all trematode species combined, and the greatest prevalence was found in the *North* and *Source* subregions (Table 1). For fish‐using trematodes, prevalence was significantly higher in the native versus introduced region, where the two species (*LS* and *ZL*) were collectively 20× less common. In contrast, bird‐using trematodes (*AV* and *HQ*) had slightly higher prevalence in the introduced region.

We also noted clear differences in haplotype diversity between bird‐ and fish‐using trematodes (Figure 3). Though bird‐using trematodes had significantly (Χ^2^ = 5.070, *p* = .024) lower haplotype richness in the introduced versus native ranges, the pattern was stronger (Χ^2^ = 29.790, *p* < .00001) for fish‐using trematodes. Haplotype frequencies demonstrated bird‐using trematodes to have relatively equal frequencies across native (25%), shared‐native (28%), shared‐introduced (30%), and introduced (17%) groupings; in contrast, fish‐using trematodes showed substantially greater frequencies in the native (67%) and shared‐native (24%) groupings compared to the shared‐introduced (5%) and introduced (5%) groupings. Moreover, in rarefaction analyses, bird‐using trematodes demonstrated less difference between predicted introduced haplotype richness and *Source* haplotype richness, when compared to fish‐using trematodes (Figure S4).

### 4.3 Genetic differentiation and gene flow

**Figure 5:**
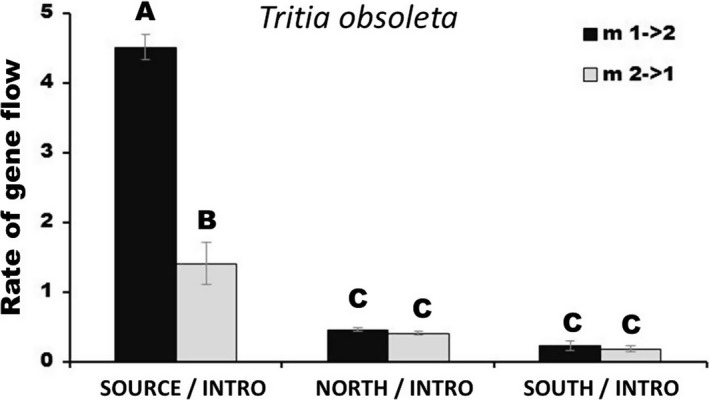
Migration rates among native subregions and the introduced region for *Tritia obsoleta*. This demonstrates the rate of gene flow based on marginal peak probabilities using the isolation with migration model (IMa) (Hey & Nielsen, 2007). Migration rates are presented as region 1 → region 2 (black) or region 2 → region 1 (gray). For example, in the first comparison, the black bar represents gene flow from the Source subregion to the introduced region, while the gray bar represents gene flow from the introduced region to the Source subregion. Significant post hoc comparisons are represented as letters above the bars. The results suggest strong directional flow from the Source subregion to the introduced region, with little evidence of gene flow to the introduced region from the North and South subregions. Other intra‐ and inter‐regional comparisons for *T. obsoleta* and also the parasite *HQ* can be found in Figure S6

To uncover the most connected subregions and populations across the ranges and pinpoint origins of gene flow to the introduced region (Hypothesis 3), we analyzed differentiation patterns and rates of migration for the snail and its trematodes. For both genetic markers, little genetic differentiation was detected for the snail host in regional comparisons. However, a few cases of differentiation were identified in the subregional analyses, primarily comparisons including the *South* subregion. The *North* subregion also demonstrated significant differentiation with some subregions, particularly *SFB* and *South*. In contrast, the *Source* subregion demonstrated few instances of significant differentiation among all regional and subregional comparisons; notably, no occurrences of significant differentiation were found for any of the comparisons between the *Source* and introduced subregions. For the parasite *HQ*, no significant differentiation was detected for regional comparisons in either genetic marker, and there were few significant comparisons among the subregions. Altogether, the host snail and its parasite demonstrated congruent patterns for limited genetic differentiation between the *Source* subregion and the introduced region. In population‐level comparisons (i.e., pairwise FSTs), we found clear associations between the introduced populations and those in the native *Source* (Figure S5A). In contrast, there were no clear patterns in population relatedness for *HQ* (Figure S5B). To complement analyses of population structure, genetic relatedness, and geographic richness/frequency data described above, we analyzed migration rate patterns for the host snail and its parasite *HQ* (using MCMC sampling in an isolation with migration framework; Hey & Nielsen, 2007). For *T. obsoleta*, strong directional gene flow was revealed from the *Source* subregion to the introduced region (Figure 5) and also to specific introduced subregions (particularly *SFB*) (Figure S6A). We also detected strong bidirectional gene flow among subregions within the introduced region, and in fact, gene flow was highest in the introduced subregions versus all other subregions. In the native subregions, there was also moderate bidirectional gene flow between the *North* and *Source*, much less gene flow between the *Source* and *South*, and negligible gene flow between the *North* and *South*. For *HQ*, patterns were not as clearly defined, in that gene flow was bidirectionally strong in all three comparisons (Figure S6B).

## 5 DISCUSSION

When species successfully colonize new regions through natural or anthropogenic transport vectors, a multitude of factors can influence resulting genetic diversity in the novel range. In situations where migrating species have diverse source pools, high propagule pressure, and relatively simple life cycles, there may be little to no perceptible bottleneck in founding locations. Consistent with this so‐called genetic paradox (Roman & Darling, 2007), the introduced host snail *T. obsoleta* demonstrated no obvious genetic bottleneck in its introduced west coast region, especially when compared to its purported *Source* region (in and around the US Mid‐Atlantic; Figure 3). In contrast, *T. obsoleta's* four trematodes collectively revealed significantly lower genetic diversity in the introduced versus native regions, though this was strongly affected by the trematode's final host taxa (fish or bird). Although host and parasite were likely introduced together, they clearly demonstrate differential genetic diversity in founding populations, with the parasites showing much greater conformity to genetic founder effects, in which genetic diversity is severely depressed in the colonized range (Barton & Charlesworth, 1984; Grosberg & Cunningham, 2001; Holland, 2000). These host–parasite differences are likely driven by the mechanisms detailed in Figure 1, including differences in source diversity, inoculum size, number of introduction events, life cycle complexity, and availability of suitable hosts. In the following sections, we discuss mechanisms leading to differential genetic diversity in the native and introduced regions for the host versus parasites that are specifically related to transfer dynamics of invasion. We then describe the strong influence of parasite life cycles and host availability on the genetic diversity patterns exhibited by the parasites, which uniquely separates them from their host dynamics. Finally, we discuss implications related to genetic structure, gene flow, and geographic connections for both the host and its parasites.

### 5.1 Transfer dynamics of host and parasite

*Tritia obsoleta* was introduced to the North American west coast as a hitchhiker with commercial oyster (*C. virginica*) shipments, which were transferred at a massive scale from 1869 to 1940 (Carlton, 1992; Miller, 2000). Oysters were harvested by dredging, a highly unselective extraction method, capturing the target species and also numerous associated organisms and sediments, including *T. obsoleta* (Ingersoll, 1881; Carlton, 1992). Live oysters were packed in barrels on refrigerated cars for transcontinental rail shipment to maximize in‐transit survival and subsequent sale in markets or to support aquaculture efforts (Ingersoll, 1881), with associated biota enjoying similar in‐transit benefits (Miller, 2000). These sustained oyster translocations would have enhanced propagule supply of *T. obsoleta* to the west coast during this time. Adding to this heavy propagule pressure is the fact that *T. obsoleta* is a highly abundant snail in its native range, with densities reaching greater than 600 snails/m^2^ in some locations where oysters were targeted for extraction (e.g., Long Island Sound; Appendix S1A). Additionally, the snail's large native source pool contains widespread genetic diversity; for example, in our study, native haplotype richness estimates were upwards of 600 haplotypes (200 in the putative *Source*; Figure S4). Consequently, a large number of diverse individuals harvested from a widespread area over a long period of time with limited mortality during transport would have resulted in the transfer of a highly diverse assemblage of *T. obsoleta* to the west coast. This would have lessened the extent of a genetic bottleneck in the region (Roman & Darling, 2007), likely contributing to the species’ establishment success in the introduced range, along with other favorable life history characteristics such as broad temperature and salinity tolerances and a generalist feeding strategy (Fofonoff et al., 2018). In fact, in its introduced range, *T. obsoleta* is now the most abundant gastropod on soft‐sediment habitats in *SFB* (Fofonoff et al., 2018).

The snail's introduction vector would have also conveyed a broad distribution of snail sizes/ages, including those more likely to be infected by trematode parasites (i.e., infection tends to increase with size due to greater contact likelihood over time; e.g., Byers et al., 2008). This factor, along with the snail's aforementioned high propagule pressure, resulted in the successful translocation of several (5/9) of the snail's native parasites to the introduced range. This relatively high parasite richness in an introduced range is in contrast to many other species introductions that are associated with much lower parasite species diversity in non‐native ranges (Blakeslee, Fowler, & Keogh, 2013; Torchin, Lafferty, Dobson, McKenzie, & Kuris, 2003). For example, the rough periwinkle snail, *Littorina saxatilis*, demonstrates twice the amount of escape from parasites in *SFB* as *T. obsoleta*, and its introduction vector (live baitworm trade) is associated with much lower propagule pressure (Blakeslee et al., 2012). Thus, though the high propagule pressure characterized by the oyster vector enhanced parasite transfers to the west coast with *T. obsoleta*, genetic diversity in these introduced parasites is still significantly lower than the native range compared to their snail host (Figure 3).

Stronger founder effect signatures in the parasites are likely due to additional steps or factors in parasite colonizations that influence the transfer of genetic diversity to a novel region. One such factor is the proportion (or prevalence) of infected hosts entrained in the introduction vector, which will depend on natural infection levels within *Source* sites. Site‐to‐site prevalence of trematode infection in snails can vary substantially along coastlines for a variety of reasons, but abundance of final hosts has been found to be critical (Byers et al., 2008; Fredensborg, Mouritsen, & Poulin, 2006; Smith, 2001). In our study, prevalence ranged from 1% to 66% in the native *Source*, with some sites only 1 km apart. Such site‐to‐site differences could be influenced by final host “hotspots,” where final hosts are attracted to a site for a specific reason, such as nesting, food, and shelter (Byers, Holmes, & Blakeslee, 2016; Smith, 2001). High prevalence *Source* sites would naturally have a greater probability of introducing infected individuals to a founding region. Another potential factor affecting the transfer of parasitized individuals and subsequent parasite genetic diversity is related to the invasion pathway; that is, the stress incurred during transport that may enhance mortality in infected individuals. Several studies have found abiotic stressors, such as temperature, can lead to differential mortality in infected versus uninfected hosts (e.g., Lafferty & Holt, 2003). However, because oysters were transported in a manner ensuring survival, these stressors may have been lessened in this particular introduction vector (Miller, 2000).

The results of our study show a number of commonalities regarding introduction vector and mechanism to a couple prior studies examining the influence of invasion on host and parasite genetic diversity. In one study, genetic diversity was investigated in a snail (*B. attramentaria*) and its trematode parasites introduced from Japan to California (Miura et al., 2006). Genetic data were used to help pinpoint source locations for *B. attramentaria's* introduction, which, like *T. obsoleta,* was also via an oyster introduction vector. Further, Miura et al. (2006) identified clear differences between two introduced trematode species in terms of final hosts and dispersal mechanisms. One trematode demonstrated clear signatures of genetic founder effects, and it was revealed that this parasite was introduced to California with its snail host. In contrast, a second trematode showed no genetic bottleneck, which was hypothesized to be the result of natural dispersal to California via migratory bird hosts. Parasite life cycle and host usage differences were additional explanations for the genetic dissimilarities between the two introduced trematode species. Likewise, in our study, we found clear disparities in trematode diversity among the four trematode species in the introduced versus native ranges that are likely attributable to life cycle differences, particularly the usage of bird versus fish final hosts (discussed further below). However, like the Miura et al. (2006) study, it is also possible that the lessened genetic bottleneck we observed for the two bird‐using trematodes in our study represents some level of natural colonization via migratory birds from the Atlantic to the Pacific coast, along with other likely mechanisms (e.g., host availability and life cycle differences). In a second study of host and parasite genetic diversity, genetic data were used to help resolve the cryptogenic status (i.e., native or non‐native status uncertain) of a common periwinkle snail host (*L. littorea*) and a prevalent trematode parasite (Blakeslee et al., 2008). Both host and parasite demonstrated congruent genetic signatures of founder effects in the non‐native (eastern North America) versus native (Europe) ranges of the host and its parasite. This trematode also uses birds as its final host and, like its host, demonstrates little genetic structure in its native and non‐native ranges, likely assisted by the dispersal of its final host.

### 5.2 Influence of parasite life cycles on host and parasite genetic diversity

Trematodes have indirect life cycles that include >1 host, and the majority of *T. obsoleta's* trematodes require three hosts to sexually reproduce (Figure S1). Thus, for a trematode to remain extant in a region, all three hosts must be present and in sufficient abundance. Trematodes with truncated life cycles may therefore have an edge by requiring fewer hosts; for example in this study, the trematode *AV* has just two hosts: *T. obsoleta* and wading birds (Blakeslee et al., 2012; Curtis, 1997; Grodhaus & Keh, 1958). While *AV* is a rare trematode in the native range, it is actually more prevalent in the introduced range (Table 1), suggesting that its less complex life cycle may have facilitated its successful colonization of the west coast. In fact, both *AV* and the other bird‐using trematode, *HQ*, demonstrated a lessened genetic bottleneck in the introduced range, when compared to the fish‐using trematodes (Figures 3 and S4). In past work, final hosts have been shown to strongly influence parasite prevalence and genetic diversity in intermediate hosts. For example, Byers et al. (2008) found that among a variety of possible drivers of parasite prevalence in a New England intertidal snail, bird final host abundance was the most important. Similarly, Hechinger and Lafferty (2005) and Fredensborg et al. (2006) found strong positive correlations between trematode and bird diversity in California tidal wetlands and soft‐sediment intertidal bays of New Zealand, respectively.

Even if a parasite species is transferred to a new location, it cannot survive, reproduce, and successfully establish without its complement of suitable hosts. Typically, host specificity in trematodes is high in the first‐intermediate stage but becomes less specific in subsequent downstream hosts (Rohde, 2005). For example, *T. obsoleta* is the obligate host for all nine of its trematode species, but that specificity is lessened at the second‐intermediate stage. Depending on the trematode, a variety of hosts within a taxonomic group could be used (e.g., polychaetes, gastropods, bivalves, nearshore fish, shrimp, crabs and other crustaceans). The final host stage is even less specific, typically utilizing a wide variety of hosts in a large taxonomic group, such as shorebirds or predatory fish (Rohde, 2005; Figure S1). An examination of the availability and types of suitable hosts utilized by *T. obsoleta's* four introduced trematodes revealed significantly more suitable bird final hosts than fish final hosts in the introduced west coast range (Blakeslee et al., 2012). In fact, *AV* and *HQ* (the two bird‐using trematodes) had a full complement of suitable hosts in all three west coast bays and were the only two trematodes detected in all three bays; in contrast, neither *LS* nor *ZL* (the fish‐using trematodes) were detected in either of the two northern bays (*WB* and *BB*) (Table 1, Figure 2). Further, it was revealed that of the identified fish final hosts for *LS* and *ZL*, very few were shared between the Atlantic and Pacific coasts of North America, whereas a number of identified bird final hosts are found in common in the two ranges (particularly gulls, a frequent final host of many trematode species) (Blakeslee et al., 2012). For these reasons, the availability of suitable host species has likely had a strong influence on the genetic diversity of *T. obsoleta's* trematode parasites, which is especially apparent in the fish‐using trematodes. This is noteworthy because *LS* and *ZL* are the most prevalent trematodes in the native east coast range with a collective average prevalence of 9% in the *Source*, which is three times higher than the collective average prevalence of *AV* and *HQ* in that same subregion. In contrast, average prevalence of *AV* and *HQ* in the introduced range is two times higher than *LS* and *ZL* (Table 1). While these latter trematodes are clearly completing their life cycles utilizing west coast fauna, they may be lacking in the variety of suitable hosts of their native range, which may be driven by less overlap in genetic similarity between the east and west coast fauna (Blakeslee et al., 2012). Such genetic differences in bird‐ versus fish‐using trematodes have also been documented in a system in the eastern Atlantic, where a native trematode species using a less vagile final host (fish) had greater genetic structure than a trematode using birds as final hosts (Feis et al., 2015). In our study, we did not see clear differences in native genetic structure among the four trematode species (i.e., all four showed low genetic structure in the native range, discussed further below); however, we did see clear genetic differences between the bird‐using and fish‐using trematodes in the non‐native range (Figure 3).

### 5.3 Genetic structure, gene flow, and geography

There was little detectable genetic structure for *T. obsoleta* throughout its native and non‐native ranges on both coasts of North America, aside from some differences observed in the *South* subregion (Table S1). For the snail's native range, this result may have been anticipated because genetic structure has been tied to reproductive strategy for numerous marine and estuarine organisms; that is, species with planktonic larvae exhibit lessened genetic structure than those with direct development strategies (e.g., Kelly & Palumbi, 2010). *Tr* *itia obsoleta* lays egg capsules that hatch into veliger larvae that spend 10–22 days in the water column before settling onto the intertidal/shallow subtidal benthos (Scheltema, 1962). Our results for native range *T. obsoleta* are therefore consistent with other marine organisms that possess larvae that spend some time in the plankton, where genetic structure is low and genetic diversity is high. For the snail's non‐native range, however, the observed high genetic diversity and low genetic structure are likely the result of a diverse source pool being transferred to the west coast over a sustained period of time (discussed above), along with the possibility for some present‐day gene flow among the subregions (discussed below).

When compared to their snail host, *T. obsoleta's* four trematode species (as examined here: *AV*, *HQ*, *LS*, *ZL*) similarly demonstrated limited genetic structure in the native range, but lower genetic diversity than *T. obsoleta* (Figure S4). These results differ from another study of snail host and trematode parasite genetic diversity (Keeney, King, Rowe, & Poulin, 2009), where the host's genetic structure was higher and its genetic diversity was lower than the host's trematode parasite. This was attributed to the direct developing reproductive strategy of the host and strong dispersal by bird definitive hosts for the trematode. Indeed, this latter explanation (final host dispersal) is supported by several studies that have found genetic structure in parasites to be strongly linked to parasite life cycles and the dispersal of hosts among variable environments. For example, Criscione, and Blouin, (2004) and Blasco‐Costa and Poulin (2013) demonstrated lower genetic structure and higher gene flow in parasite species that utilized hosts, or had life cycles, that moved among habitats or environments (allogenic) compared to those that remained within the same habitat or environment (autogenic). Further, a study by Blasco‐Costa, Waters, and Poulin (2012) found that both biotic (definitive host) dispersal and abiotic (river flow) dispersal strongly influenced the resulting genetic structures and gene flow of two species of trematode parasites. Our study is likewise supportive of this link between genetic structure and life cycles for *T. obsoleta's* four trematode species, which move among multiple hosts, habitats, and geographic regions during their life cycles, especially via their more dispersive final hosts.

When we compared both host and trematode diversity in the native range to the introduced range, multiple analyses demonstrated strong connections to the *Source* and *North* subregions (Figures 2, S2, and S5), which supports historical evidence of the Mid‐Atlantic and southern New England as likely sources for *T. obsoleta's* introduction, given massive oyster transplantation over decades from these areas (Miller, 2000). Further, in an nMDS plot, most of the spatially (and genetically) closest populations to the west coast were from the *Source* subregion (Figure S5). Indeed, strong directional gene flow from the *Source* to the introduced region was detected in our gene flow analyses with very little gene flow coming from the other two native subregions (Figure 5), again supporting historical evidence pinpointing the Mid‐Atlantic as the likely source. Interestingly, predicted gene flow rates were actually highest in intraregional comparisons in the introduced region (Figure S6), suggesting strong connections among the west coast bays. Although these bays are discrete and isolated from one another (Figure 2), it is possible that undetected populations of *T. obsoleta* may exist in between the bays and contribute to the strong gene flow detected among them, particularly since the snail has been reported in other west coast populations (although these have not been identified as established) (Fofonoff et al., 2018). Alternatively, or in addition, this high rate of gene flow could be the result of current‐driven dispersal between and among bays via the snail's veliger larvae. Indeed, current‐driven dispersal has likely led to the continued spread of another prominent non‐native species on the west coast, *C. maenas* (reviewed in Fofonoff et al., 2018). Moreover, human‐mediated dispersal via shipping is another possible mechanism since shipping is a prominent anthropogenic vector for numerous marine organisms (Seebens, Gastner, Blasius, & Courchamp, 2013). Finally, it is possible that the high west coast gene flow detected in our analyses could be an artifact of the snail's recent introduction to the west coast bays and the more genetic similarity among them, especially when compared to much lower intraregional gene flow among the east coast subregions. Greater surveying along the west coast for previously undetected *T. obsoleta* populations, as well as sampling for larval and human‐mediated dispersal between bays, could help determine the most likely mechanisms influencing *T. obsoleta* gene flow and spread in the west coast.

Because sampling effort will influence observed genetic diversity in populations, we used rarefaction analyses to estimate expected genetic diversity in each region. Our results suggested that the expected asymptote in haplotype richness was substantially higher for the whole native region compared to the purported *Source* subregion (Figure S4). This is a likely outcome since the native region is quite broad and will accrue a greater amount of diversity with increasing area (Struebig et al., 2011). Interestingly, however, expected total richness in the introduced region was about two times greater than the *Source* subregion, suggesting that other populations outside our prescribed *Source* subregion may have contributed diversity to the west coast—indeed, several of our analyses pointed toward the *North* subregion as being another important contributor to west coast diversity. It is also quite possible that we missed sampling important east coast populations that may have contributed to the west coast diversity. In fact, a number of introduced haplotypes for *T. obsoleta* (*n* = 54) and its trematodes (*n* = 30) were not detected in our east coast sampling. As hypothesized in other species introductions (e.g., Blakeslee et al., 2008; Miura et al., 2006; Roman, 2006), this is likely a sampling artifact given the broad diversity of the native range (Figure S4). A less parsimonious explanation is that these unshared haplotypes represent new diversity that has accrued in the last ~100 years since the host–parasite introduction to the west coast. Particularly for the genes used as markers in this study, it would take much longer for the diversity of new mutations to occur within this time frame (Blakeslee et al., 2008; Miura et al., 2006).

### 5.4 Detection of cryptic taxa

During phylogenetic analyses, we detected genetically distinct cryptic taxa in two trematode species, *HQ* and *LS* (Figures 4 and S3). These lineages were also found in the introduced range, and for *HQ*, two were also found with the 18S marker. Cryptic taxa are an increasingly common discovery in trematode phylogenetics. For example, Huspeni (2000) showed that one trematode species (*Parorchis acanthus*) was actually four genetically distinct species, while Miura et al. (2005), Miura et al. (2006) discovered as many as 10 distinct lineages in a single described species of trematode in *Batillaria cumingi*. Similarly, Leung, Keeney, and Poulin (2009) found four genetically distinct trematode species previously described as one. Trematodes are typically identified using keys, plates, and images, but morphological characters (especially those associated with genetic differences) can be difficult to detect. Our discovery further highlights that trematodes are a diverse group with underdeveloped taxonomic, ecological, and evolutionary knowledge. In the future, there is much to learn about the cryptic lineages detected in this study, including whether morphological distinctions can be found, whether they infect or influence the snail host differently, and whether they utilize different downstream hosts. Further research can help reveal the role that these cryptic taxa play, considering differential roles and invasion histories of cryptic trematodes found in other studies (e.g., Miura et al., 2006).

## 6 CONCLUSIONS AND SIGNIFICANCE

Our study reveals clear distinctions between hosts and parasites in resulting genetic diversity and the detection of genetic bottlenecks following colonization events, and the strong influence of parasite life cycles on parasite genetic diversity (e.g., vagility and distribution of final hosts). Most prior work on founder effects in species introductions has focused on free‐living species; thus, our study enhances understanding regarding the mechanisms that drive genetic diversity losses in founding populations in both free‐living and parasitic species. As many introduction vectors continue to operate and transport species to new locations around the world, understanding the mechanisms that influence genetic diversity in founding populations can be informative for a multitude of systems. Future work can help resolve the many unanswered and untested questions that remain in this and other host–parasite systems, and further reveal the important role that cryptic species such as parasites play in systems around the globe.

## Data Availability

All data are included with the manuscript as tables or figures in the main text, or as tables or figures in the Supporting Information and Appendices. Sequences have been deposited into GenBank (https://www.ncbi.nlm.nih.gov/genbank/) as accession numbers: MN272433–MN272597 and MN272598–MN272732.
