# Supplementary material for: Founder effects and species introductions: A host versus parasite perspective
Source: Evol Appl. 2019 Sep 26;13(3):559–74. doi: 10.1111/eva.12868 (PMC7045715; doi:10.1111/eva.12868)
Supplement: Supplementary file 1 [file EVA-13-559-s001.docx]

**SUPPORTING INFORMATION**

**Table S1. Primer information for host and trematodes.**

| **Species** | **Marker** | **Fragment**  **Size (bp)** | **Forward Primer** | **Reverse Primer** | **Root Species (Genbank**  **Accession Number; Citation)** |
| --- | --- | --- | --- | --- | --- |
| *Tritia obsoleta* | COI | 546 | TCGTGCTGAACTTGGACAAC | CCCCAGCTAATACAGGCAAA | *Cyclope neritea* (AY789981.1;  Simon-Bouhet et al., 2006) |
| *Tritia obsoleta* | 18S | 654 | AAACGGCTACCACATCCAAG | TGGCATCGTTTATGGTCAGA | *Crepidula fornicata* (AY377660.1;  Okusu et al. 2003) |
| *Austrobilharzia variglandis* | COI | 571 | CGCCTCTGTCTGTTGTTGAA | AAACCCCAACACTCACCAAA | *Fasciola hepatica* (X15613.1;  Garey et al. 1989) |
| *Himasthla quissitensis* | COI | 522 | CTGCGTCGGTTTGTTTAGGT | TCCCCAAACACACAATAGCC | *Fasciola hepatica* (X15613.1;  Garey et al. 1989) |
| *Himasthla quissitensis* | 18S | 539 | GCGAATGGCTCATTAAATCAG | CTTGTTACGACTTTTACTTCC | *Fasciola hepatica* (AJ004969.1;  Fernandez et al. 1998) |
| *Lepocreadium setiferoides* | COI | 514 | CCCCCTTGTCGAGTGGGGAT | TGCAGTATGCACATCCAAACCCACC | *Fasciola hepatica* (X15613.1;  Garey et al. 1989) |
| *Zoogonus lasius* | COI | 535 | CCGCCTTTATCTTCTGTGGA | TATGCACATCCAAACCAACC | *Fasciola hepatica* (X15613.1;  Garey et al. 1989) |

Listed are the various primer pairs used to amplify the cytochrome oxidase I (COI) gene fragment or the 18S ribosomal gene fragment for the host snail, *Tritia obsoleta*, and the four trematode parasites included in the study. Also included are the species and accession number used to root phylogenetic trees. The overlapping region of the COI fragment for all four trematodes was 460 bp.

**Table S2. Fixation indices of regional and subregional differentiation following AMOVA in *Tritia obsoleta* (host) and *Himasthla quissitensis* (parasite) for COI and 18S markers.**


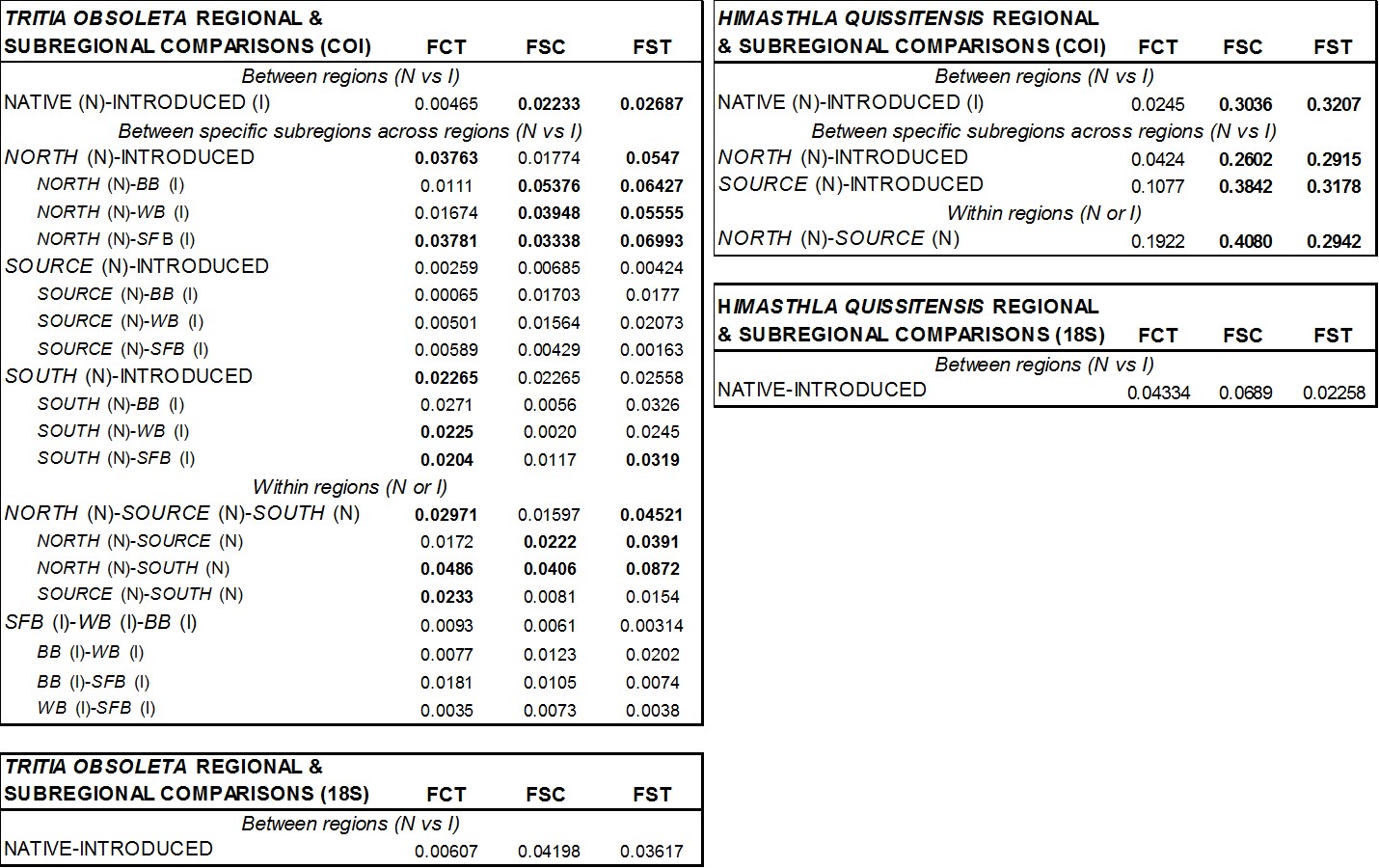


FCT=fixation index among groups; FSC=fixation index among populations within groups; FST=fixation index within populations. Bolded values represent significant differentiation for comparisons.


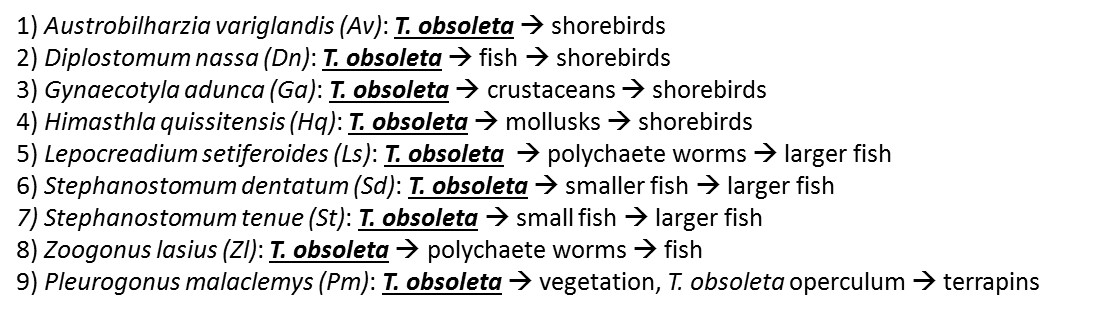


**Fig. S1.** This figure depicts the nine trematode species described in *Tritia obsoleta* and their respective second-intermediate and final hosts (major taxa; see Blakeslee et al., 2012 and Phelan et al., 2016 for more detailed information on host species). Second-intermediate hosts can include a wide variety of invertebrates and vertebrates. Definitive hosts are primarily split between fish and shorebirds, with one species utilizing terrapins as its final host. Five of these species have been detected in western North America: AV, HQ, LS, SD, and ST. In our study here, SD had too few samples to analyze for genetic diversity in both regions.

**
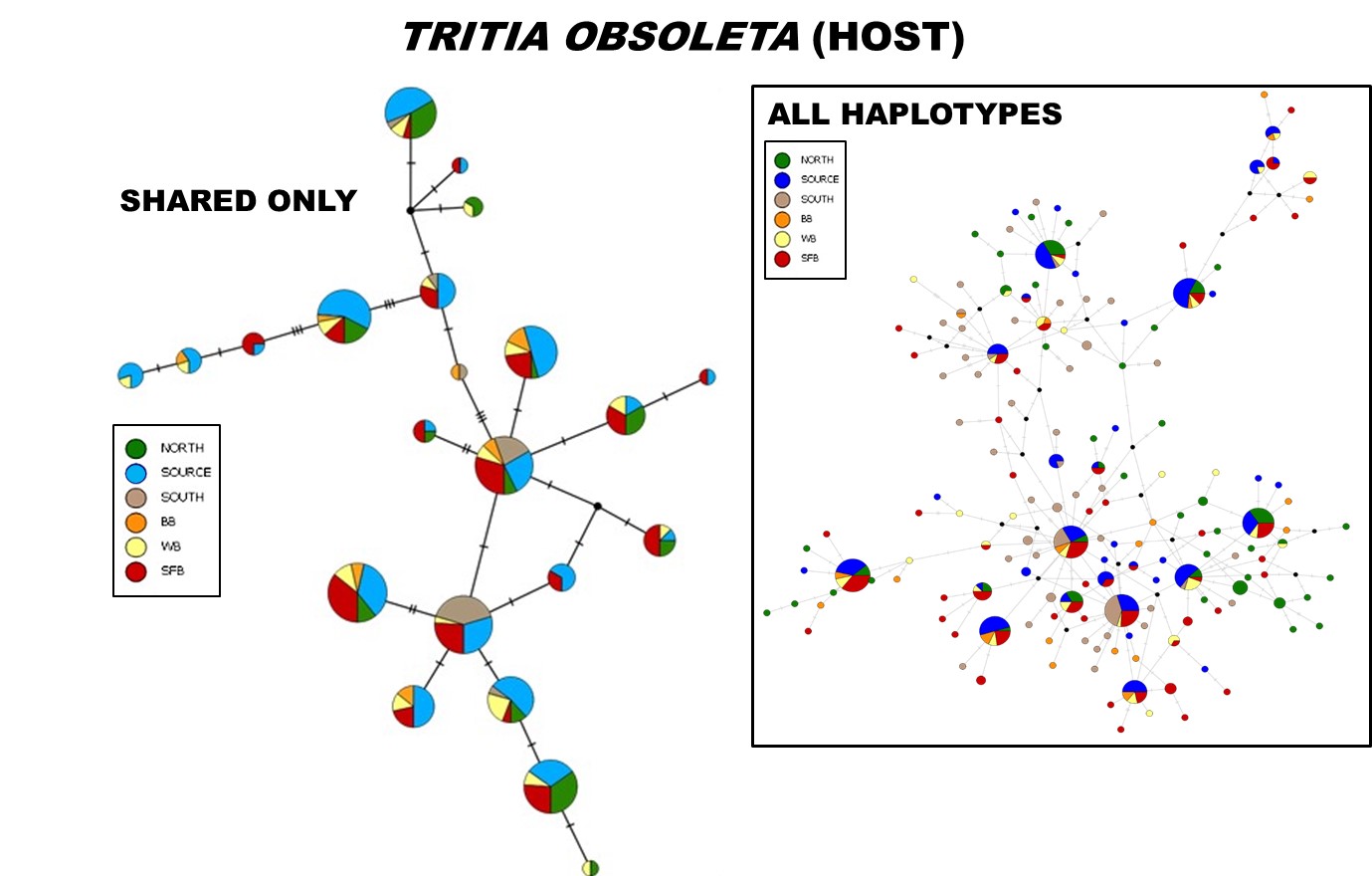
**

**Fig. S2. Haplotype network (COI) for *Tritia obsoleta*.** Haplotype connections are depicted for the shared haplotypes in the two major regions. The size of circles is scaled to the number of occurrences for each haplotype. Colors represent different subregions in the native and introduced regions (see Appendix B). The inset represents all the haplotypes detected during our study, including a considerable number of unshared haplotypes.


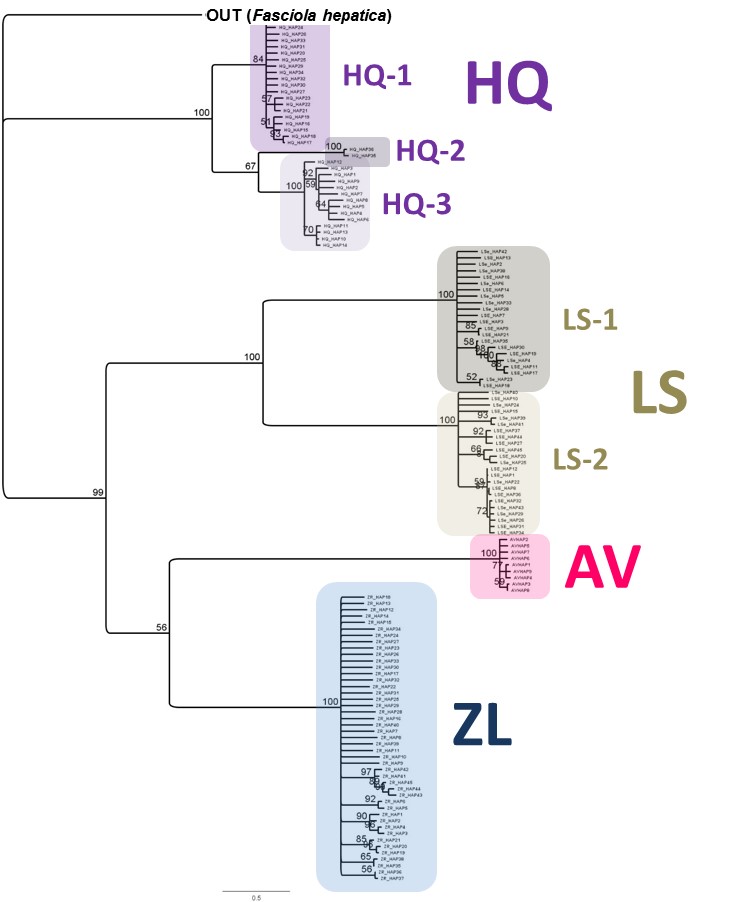


**Fig. S3. Bayesian phylogenetic tree (COI maker) for the four trematode species**. Phylogenetic tree performed using Mr. Bayes 3.2.6. Shaded colors represent genetically distinct lineages based on the consensus tree.


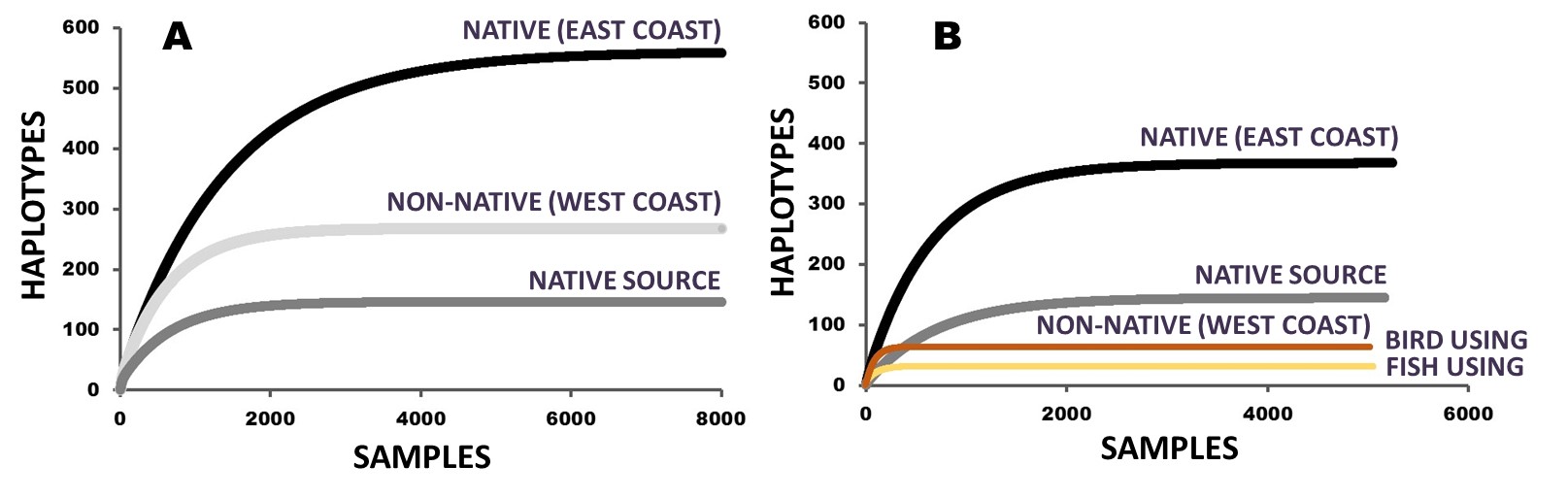


**Fig. S4. Haplotype accumulation curves (COI)** for (A) the host snail, *Tritia obsoleta*, and (B) four of its trematode species (*Austrobilharzia variglandis, Himasthla quissitensis, Lepocreadium setiferoides, Zoogonus lasius*) on both coasts of North America. The trematodes are also broken into bird-using and fish-using groups for their definitive hosts (B).


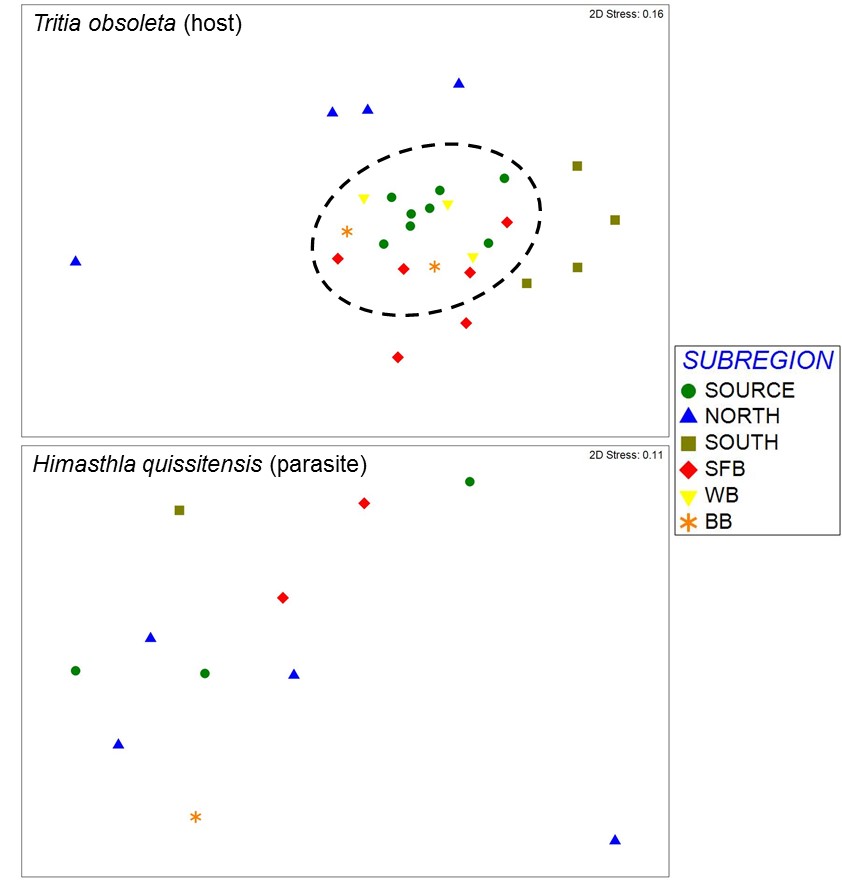


**Fig. S5. nMDS plots of pairwise FST data (COI marker) for *Tritia obsoleta* and *Himasthla quissitensis*.** Symbols and colors represent subregions in *Native* and *Introduced*. Samples contained within the hashed oval indicate sites that were not significantly differentiated from one another in pairwise analyses.


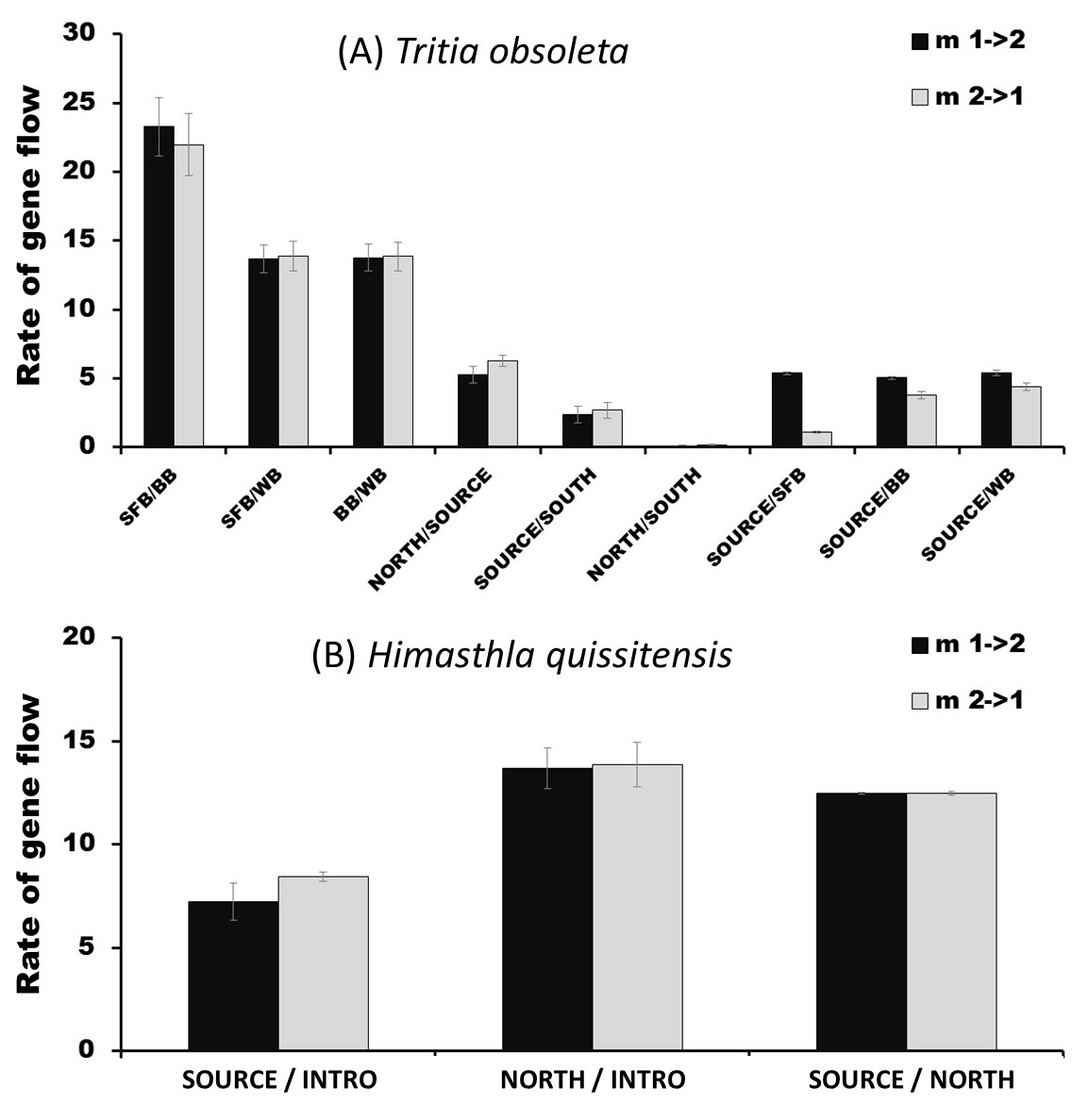


**Fig. S6. Migration rates among regions and subregions for *Tritia obsoleta* and *Himasthla quissitensis*.** Per Fig. 5, migration rates are presented as region 1 🡪 region 2 (black) or region 2 🡪 region 1 (gray). (A) *T. obsoleta* subregions in native and introduced regions, where the *Source* region is highlighted for inter-subregional comparisons given the results of Figure 5 showing the strongest gene flow from the *Source* to the introduced region. (B) Rate of gene flow for *HQ* for regional and subregional comparisons. For *HQ*, we only explored three comparisons given limited sample size in some subregions.
