# Supplementary material for: Founder effects and species introductions: A host versus parasite perspective
Source: Evol Appl. 2019 Sep 26;13(3):559–74. doi: 10.1111/eva.12868 (PMC7045715; doi:10.1111/eva.12868)
Supplement: Supplementary file 2 [file EVA-13-559-s002.docx]

**APPENDICES**

**APPENDIX A: *Tritia obsoleta* densities in native and introduced sites (this study) based on random quadrat (0.25 m^2^) sampling along a 30-m transect line.**

| Site | Region (I/N) | Date | Quadrat 1 | Quadrat 2 | Quadrat 3 | Quadrat 4 | Quadrat 5 | Quadrat Average | Snails / m^2^ |
| --- | --- | --- | --- | --- | --- | --- | --- | --- | --- |
| Tappen Beach Park, Sea Cliff, NY | N | 9/14/2012 | 36 | 111 | 298 | 299 | 111 | 171 | 684 |
| Tappen Beach Park, Sea Cliff, NY | N | 10/15/2012 | 32 | 48 | 1 | 2 | 0 | 16.6 | 66.4 |
| Cape Henlopen Fishing Pier, Lewes, DE | N | 11/1/2010 | 2 | 55 | 10 | 13 | 0 | 16 | 64 |
| Route 101, WA (algae) | I | 7/14/2010 | 5 | 4 | 2 | 3 | 6 | 4 | 16 |
| Route 101, WA (oysters) | I | 7/14/2010 | 22 | 5 | 4 | 11 | 0 | 8.4 | 33.6 |
| Nahcotta, WA (algae) | I | 7/13/2010 | 0 | 0 | 0 | 0 | 0 | 0 | 0 |
| Nahcotta, WA (oyster) | I | 7/13/2010 | 11 | 0 | 2 | 1 | 0 | 2.8 | 11.2 |
| N. Nemah Rd., South Bend, WA | I | 7/14/2010 | 4 | 4 | 11 | 5 | 7 | 6.2 | 24.8 |
| Blackie Spit, Surrey, BC (10 m from shore) | I | 7/15/2010 | 11 | 20 | 24 | 6 | 7 | 13.6 | 54.4 |
| Blackie Spit, Surrey, BC (50 m from shore) | I | 7/15/2010 | 48 | 13 | 2 | 8 | 21 | 18.4 | 73.6 |
| Elgin Heritage Park, Surrey, BC | I | 7/15/2010 | 86 | 81 | 74 | 72 | 69 | 76.4 | 305.6 |
|  |  |  |  |  |  |  |  |  |  |

**APPENDIX B: *Tritia obsoleta* COI hapolotype occurrences and site totals in the native east coast and introduced west coast.** Sites in the *North* include: (1) Harpswell (HAR), Maine; (2) Portland (POR), Maine; (4) Ipswich (IP), Massachusetts; and (3) Jackson Estuarine Lab (JEL), Durham, New Hampshire. Sites in the *Source* include: (5) Mystic (MYS), Connecticut; (8) Port Jefferson (PJ), New York; (7) Wading River (WR), New York; (6) Goldsmith (GS), New York; (9) Shinnecock (SHIN), New York; (10) Lewes (LEW), Delaware; (12) Virginia Beach (VB), Virginia; and (11) Wachapreague (WACH), Virginia. Sites in the *South* include: (13) Wilmington (WNC), North Carolina; (15) Ace Basin (AB), South Carolina; (14) North Inlet (NI), South Carolina; and (16) Savannah (GA), Georgia. Sites in *BB* include: (18) Blackie Spit (BLS), Surrey, British Columbia, Canada; and (17) Elgin Heritage Park, Surrey, British Columbia. Sites in *WB* include: (19) South Bend (SB), Washington; (20) Nahcotta (NAH), Washington; and (21) Route 101 (101), Washington. Sites in *SFB* include the following: (27) Coyote Marina (COY), California; (22) MLK Shoreline, Oakland, California; (23) Bay Farm Island (BFI), California; (24) San Leandro (SL), California; (25) Hayward (HAY), California; and (26) Redwood City Marina (RWC), California. Red highlighting represents haplotypes only found in the west coast; green highlighting represents haplotypes only found in the east coast; no highlighting (white) represents shared haplotypes.

| **Hap** | **North American East Coast** | | | | | | | | | | | | | | | | | **North American West Coast** | | | | | | | | | | | |
| --- | --- | --- | --- | --- | --- | --- | --- | --- | --- | --- | --- | --- | --- | --- | --- | --- | --- | --- | --- | --- | --- | --- | --- | --- | --- | --- | --- | --- | --- |
|  | **HAR** | **POR** | **IP** | **JEL** | **MYS** | **PJ** | **WR** | **GS** | **SHIN** | **LEW** | **VB** | **WACH** | **WNC** | **AB** | **NI** | **GA** | **BLS** | | **EHP** | **SBD** | **NAH** | **101** | **COY** | **MLK** | **BFI** | **SL** | **HAY** | **RWC** |  |
| 1 | 2 | 6 |  |  | 1 | 1 | 1 |  | 2 |  |  | 2 |  |  |  |  |  | |  |  |  | 2 |  | 1 | 2 | 3 |  |  |  |
| 2 |  |  |  |  |  |  | 2 |  |  |  |  | 3 | 1 |  |  |  |  | |  |  |  | 1 |  |  | 2 | 1 |  |  |  |
| 3 |  | 2 | 2 |  | 1 | 2 | 1 | 3 | 2 |  | 3 | 1 |  |  |  |  | 1 | |  |  |  | 2 | 1 | 2 |  |  |  |  |  |
| 4 |  |  |  |  |  |  |  |  |  |  |  |  |  |  |  |  |  | |  | 1 |  | 1 |  |  |  |  |  | 1 |  |
| 5 |  | 1 |  | 2 | 1 | 1 |  | 2 |  |  | 3 | 3 |  |  |  |  | 2 | |  |  |  | 3 | 3 | 3 |  | 2 | 1 | 1 |  |
| 6 |  |  |  |  |  |  |  |  |  |  |  |  |  |  |  |  |  | |  |  |  | 1 | 1 |  |  |  |  |  |  |
| 7 | 2 | 2 | 2 | 1 | 1 | 1 | 3 | 1 | 1 | 2 |  | 1 |  |  |  | 1 |  | |  |  | 1 | 1 |  |  |  | 1 |  |  |  |
| 8 |  |  |  |  |  |  |  |  |  |  |  |  |  |  |  |  |  | |  |  |  | 1 |  |  |  |  |  |  |  |
| 9 |  |  |  |  | 2 |  |  |  |  |  | 1 | 1 |  |  |  |  |  | |  |  |  | 1 |  |  |  |  |  |  |  |
| 10 | 1 |  |  | 1 | 2 | 2 | 1 |  | 1 | 3 |  |  |  |  |  | 1 |  | |  | 1 | 2 | 1 | 1 |  |  |  |  |  |  |
| 11 |  |  |  | 1 |  |  |  |  |  |  |  |  |  |  |  |  |  | |  |  |  | 1 |  |  |  |  |  |  |  |
| 12 |  |  |  |  |  |  |  |  |  |  |  |  |  | 1 |  |  |  | |  |  |  |  |  |  |  |  |  |  |  |
| 13 |  |  |  |  |  |  | 1 |  | 2 | 2 | 2 | 1 | 5 | 2 |  | 4 |  | |  | 1 |  |  | 2 | 1 | 1 | 2 | 1 |  |  |
| 14 |  |  |  |  |  |  | 1 | 3 |  |  |  |  |  | 1 |  |  |  | |  |  |  |  |  |  |  |  |  |  |  |
| 15 |  |  |  |  |  |  |  |  |  |  |  |  |  | 1 |  |  |  | |  |  |  |  |  |  |  |  |  |  |  |
| 16 |  |  |  |  |  |  |  |  |  |  |  |  |  | 1 |  |  |  | |  |  |  |  |  |  |  |  |  |  |  |
| 17 |  |  |  |  |  |  |  |  |  |  |  |  |  | 1 |  |  |  | |  |  |  |  |  |  |  |  |  |  |  |
| 18 |  |  |  |  |  |  |  |  |  |  |  |  | 1 | 1 |  |  |  | |  |  |  |  |  |  |  |  |  |  |  |
| 19 |  |  |  |  |  |  |  |  |  |  |  |  |  | 2 |  |  |  | |  |  |  |  |  |  |  |  |  |  |  |
| 20 |  |  |  |  |  |  |  |  |  |  |  |  |  | 1 |  |  |  | |  |  |  |  |  |  |  |  |  |  |  |
| 21 |  |  |  |  |  |  |  |  |  |  |  |  |  | 1 |  |  |  | |  |  |  |  |  |  |  |  |  |  |  |
| 22 |  |  |  |  |  |  |  |  |  |  |  |  |  | 1 |  |  |  | |  |  |  |  |  |  |  |  |  |  |  |
| 23 |  |  |  |  |  |  |  |  |  |  |  |  |  | 1 |  |  |  | |  |  |  |  |  |  |  |  |  |  |  |
| 24 |  |  |  |  |  |  |  |  |  |  |  |  |  | 1 |  |  |  | |  |  |  |  |  |  |  |  |  |  |  |
| 25 | 1 |  | 1 |  |  | 2 |  | 1 | 2 | 1 |  | 1 | 2 |  | 2 | 2 | 2 | |  | 1 | 1 |  | 1 | 4 | 2 | 1 |  |  |  |
| 26 |  |  |  |  |  |  |  |  |  |  |  |  |  |  |  |  |  | |  |  |  |  |  |  | 1 |  |  |  |  |
| 27 |  |  |  |  |  |  |  |  |  | 1 | 1 | 2 |  |  |  |  |  | |  |  |  |  |  | 1 | 1 |  |  |  |  |
| 28 |  |  |  |  |  |  |  |  |  |  |  |  |  |  |  |  | 1 | |  | 2 |  |  |  | 1 | 1 |  |  |  |  |
| 29 |  |  |  |  |  |  |  |  |  |  |  |  |  |  |  |  |  | |  |  |  |  |  |  | 1 |  |  |  |  |
| 30 |  |  |  |  |  |  |  |  |  |  |  |  |  |  |  |  |  | |  |  |  |  |  |  | 1 |  |  |  |  |
| 31 |  |  |  |  |  |  |  |  |  |  |  |  |  |  |  |  |  | |  |  |  |  |  |  | 1 |  |  |  |  |
| 32 |  |  |  |  |  |  |  |  |  |  |  |  |  |  |  |  |  | |  |  |  |  |  |  | 1 |  |  |  |  |
| 33 | 1 |  |  |  |  |  |  | 1 |  |  |  |  |  |  |  |  |  | |  |  |  |  |  | 1 | 1 |  |  |  |  |
| 34 |  | 2 |  |  |  |  | 1 |  |  |  |  |  |  |  |  |  |  | |  |  | 1 |  |  |  | 2 | 2 |  |  |  |
| 35 |  |  | 1 |  | 3 | 1 | 2 | 2 | 3 |  |  |  |  |  |  |  | 3 | |  | 1 | 1 |  |  | 2 | 1 | 2 |  |  |  |
| 36 |  |  |  |  |  |  |  |  |  |  |  |  |  |  |  |  |  | |  |  |  |  |  |  | 1 |  |  |  |  |
| 37 |  | 1 | 2 | 1 | 1 |  |  |  |  |  | 1 |  |  |  |  |  |  | |  | 1 | 1 |  |  | 1 | 1 | 2 |  |  |  |
| 38 |  |  |  |  |  |  |  |  |  |  |  |  |  |  |  |  | 1 | |  |  |  |  |  |  |  |  |  |  |  |
| 39 |  |  |  |  |  |  |  |  |  |  |  |  |  |  |  |  | 1 | |  |  |  |  |  |  |  |  |  |  |  |
| 40 |  |  |  |  |  |  |  |  |  |  |  |  |  |  |  |  | 1 | |  |  |  |  |  |  |  |  |  |  |  |
| 41 |  |  |  |  | 2 | 1 | 1 | 1 |  | 2 |  |  |  |  |  |  | 1 | | 1 | 1 | 1 |  |  | 1 |  | 1 |  | 1 |  |
| 42 |  |  |  |  |  |  |  |  |  |  |  |  |  |  |  |  | 1 | |  |  |  |  |  |  |  |  |  |  |  |
| 43 |  |  |  |  |  |  |  |  |  |  |  |  |  |  |  |  | 1 | |  |  |  |  |  | 1 |  |  |  |  |  |
| 44 |  |  |  |  |  |  |  |  |  |  |  |  |  |  |  |  | 1 | |  |  |  |  |  |  |  |  |  |  |  |
| 45 |  |  |  |  |  |  |  |  |  |  |  |  |  |  | 1 |  | 1 | |  |  |  |  |  |  |  |  |  |  |  |
| 46 |  |  |  |  |  |  |  |  |  |  |  |  |  |  |  |  | 1 | |  |  |  |  |  |  |  |  |  |  |  |
| 47 |  |  |  |  |  |  |  |  |  |  |  |  |  |  |  |  | 1 | |  |  |  |  |  |  |  |  |  |  |  |
| 48 |  |  |  |  |  |  |  |  |  |  |  |  |  |  |  |  |  | |  |  |  |  | 1 |  |  |  |  |  |  |
| 49 |  |  |  |  |  |  |  |  |  |  |  |  |  |  |  |  |  | |  |  |  |  | 1 |  |  | 1 |  |  |  |
| 50 |  |  |  |  |  |  |  |  |  | 1 |  |  |  |  |  |  |  | |  |  |  |  | 3 |  |  |  |  |  |  |
| 51 |  |  |  |  |  |  |  |  |  |  |  |  |  |  |  |  |  | |  |  |  |  | 1 |  |  |  |  |  |  |
| 52 |  |  |  |  | 1 |  |  |  |  |  |  |  |  |  |  |  |  | |  |  |  |  | 1 |  |  |  |  |  |  |
| 53 |  |  |  |  |  |  |  |  |  |  |  |  |  |  |  |  |  | | 1 |  |  |  |  |  |  |  |  |  |  |
| 54 |  |  |  |  |  |  |  |  | 1 | 2 |  |  |  |  |  |  |  | | 1 | 1 |  |  |  |  |  |  |  |  |  |
| 55 |  |  |  |  |  |  |  |  |  |  |  |  |  |  |  |  |  | | 1 |  |  |  |  |  |  |  |  |  |  |
| 56 |  |  |  |  |  |  |  |  |  |  |  |  |  |  |  |  |  | | 1 |  |  |  |  |  |  |  |  |  |  |
| 57 |  |  |  |  |  |  |  |  |  |  |  |  |  |  |  | 1 |  | |  |  |  |  |  |  |  |  |  |  |  |
| 58 |  |  |  |  |  |  |  |  |  |  |  |  |  |  |  | 1 |  | |  |  |  |  |  |  |  |  |  |  |  |
| 59 |  |  |  |  |  |  |  |  |  |  |  |  |  |  |  | 1 |  | |  |  |  |  |  |  |  |  |  |  |  |
| 60 |  |  |  |  |  |  |  |  |  |  |  |  |  |  |  | 1 |  | |  |  |  |  |  |  |  |  |  |  |  |
| 61 |  |  |  |  |  |  |  |  |  |  |  |  |  |  |  | 1 |  | |  |  |  |  |  |  |  |  |  |  |  |
| 62 |  |  |  |  |  |  |  |  |  |  |  |  |  |  |  | 1 |  | |  |  |  |  |  |  |  |  |  |  |  |
| 63 |  |  |  |  |  |  |  |  |  |  |  |  |  |  |  | 1 |  | |  |  |  |  |  |  |  |  |  |  |  |
| 64 |  |  |  |  |  |  |  |  |  |  |  |  |  |  |  | 1 |  | |  |  |  |  |  |  |  |  |  |  |  |
| 65 |  |  |  |  |  |  |  |  |  |  |  |  |  |  |  | 1 |  | |  |  |  |  |  |  |  |  |  |  |  |
| 66 |  |  |  |  |  |  |  |  |  |  |  |  |  |  |  | 1 |  | |  |  |  |  |  |  |  |  |  |  |  |
| 67 |  |  |  |  |  |  |  | 1 |  |  |  |  |  |  |  |  |  | |  |  |  |  |  |  |  |  |  |  |  |
| 68 | 1 | 1 |  |  |  |  |  |  |  |  |  |  |  |  |  |  |  | |  |  |  |  |  |  |  |  |  |  |  |
| 69 | 1 |  |  |  |  |  |  |  |  |  |  |  |  |  |  |  |  | |  |  |  |  |  |  |  |  |  |  |  |
| 70 | 2 |  |  |  |  |  |  |  |  |  |  |  |  |  |  |  |  | |  |  | 1 |  |  |  |  |  |  |  |  |
| 71 | 1 |  |  |  |  |  |  |  |  |  |  |  |  |  |  |  |  | |  |  |  |  |  |  |  |  |  |  |  |
| 72 | 1 |  |  |  |  |  |  |  |  |  |  |  |  |  |  |  |  | |  |  |  |  |  |  |  |  |  |  |  |
| 73 | 1 |  |  |  |  |  |  |  |  |  |  |  |  |  |  |  |  | |  |  |  |  |  |  |  |  |  |  |  |
| 74 | 1 |  |  |  |  |  |  |  |  |  |  |  |  |  |  |  |  | |  |  |  |  |  |  |  |  |  |  |  |
| 75 |  |  |  |  |  |  |  |  |  |  |  |  |  |  |  |  |  | |  |  |  |  |  |  |  |  | 1 |  |  |
| 76 |  |  |  |  |  |  |  |  |  |  |  |  |  |  |  |  |  | |  |  |  |  |  |  |  |  | 1 |  |  |
| 77 |  |  |  |  |  |  |  |  |  |  |  |  |  |  |  |  |  | |  |  |  |  |  |  |  |  | 1 |  |  |
| 78 |  |  |  |  |  |  |  |  |  |  |  |  |  |  |  |  |  | |  |  |  |  |  |  |  |  | 3 |  |  |
| 79 |  |  |  |  |  |  |  |  |  |  |  |  |  |  |  |  |  | |  |  |  |  |  |  |  |  | 2 |  |  |
| 80 |  |  |  |  |  |  |  |  |  |  |  |  |  |  |  |  |  | |  |  |  |  |  |  |  |  | 1 |  |  |
| 81 |  |  |  |  |  |  |  |  |  |  |  |  |  |  |  |  |  | |  |  |  |  |  |  |  |  | 1 |  |  |
| 82 |  |  |  |  |  |  |  |  |  |  |  |  |  |  |  |  |  | |  |  |  |  |  |  |  |  | 1 |  |  |
| 83 |  |  |  |  |  |  |  |  |  |  |  |  |  |  |  |  |  | |  |  | 2 |  |  |  |  | 1 | 1 |  |  |
| 84 |  |  |  |  |  |  |  |  |  |  |  |  |  |  |  |  |  | |  |  |  |  |  |  |  |  | 1 |  |  |
| 85 |  |  |  |  |  |  |  |  |  |  |  |  |  |  |  |  |  | |  |  |  |  |  |  |  |  | 1 |  |  |
| 86 |  |  |  |  |  |  |  |  |  |  |  |  |  |  |  |  |  | |  |  |  |  |  |  |  |  | 1 |  |  |
| 87 |  |  |  |  |  |  |  |  |  |  |  |  |  |  |  |  |  | |  |  |  |  |  |  |  |  | 1 |  |  |
| 88 |  |  | 1 |  |  |  |  |  |  |  |  |  |  |  |  |  |  | |  |  |  |  |  |  |  |  |  |  |  |
| 89 |  |  | 1 |  |  |  |  |  |  |  |  |  |  |  |  |  |  | |  |  |  |  |  |  |  |  |  |  |  |
| 90 |  |  | 1 | 2 |  |  |  |  |  |  |  |  |  |  |  |  |  | |  |  |  |  |  |  |  |  |  |  |  |
| 91 |  |  | 1 |  |  |  |  |  |  |  |  |  |  |  |  |  |  | |  |  |  |  |  |  |  |  |  |  |  |
| 92 |  |  | 1 |  |  |  |  |  |  |  |  |  |  |  |  |  |  | |  |  |  |  |  |  |  |  |  |  |  |
| 93 |  |  | 1 |  |  |  |  |  |  |  |  |  |  |  |  |  |  | |  |  |  |  |  |  |  |  |  |  |  |
| 94 |  | 3 | 2 |  |  |  |  |  |  |  |  |  |  |  |  |  |  | |  |  |  |  |  |  |  |  |  |  |  |
| 95 |  |  | 1 |  |  |  |  |  |  |  |  |  |  |  |  |  |  | |  |  |  |  |  |  |  |  |  |  |  |
| 96 |  |  |  | 1 |  |  |  |  |  |  |  |  |  |  |  |  |  | |  |  |  |  |  |  |  |  |  |  |  |
| 97 |  |  |  | 1 |  |  |  |  |  |  |  |  |  |  |  |  |  | |  |  |  |  |  |  |  |  |  |  |  |
| 98 |  |  |  | 1 |  |  |  |  |  |  |  |  |  |  |  |  |  | |  |  |  |  |  |  |  |  |  |  |  |
| 99 |  |  |  | 1 |  |  |  |  |  |  |  |  |  |  |  |  |  | |  |  |  |  |  |  |  |  |  |  |  |
| 100 |  |  |  | 1 |  |  |  |  |  |  |  |  |  |  |  |  |  | |  |  |  |  |  |  |  |  |  |  |  |
| 101 |  |  |  | 1 |  |  |  |  |  |  |  |  |  |  |  |  |  | |  |  |  |  |  |  |  |  |  |  |  |
| 102 |  |  |  | 1 |  |  |  |  |  |  |  |  |  |  |  |  |  | |  |  |  |  |  |  |  |  |  |  |  |
| 103 |  |  |  | 1 |  |  |  |  |  |  |  |  |  |  |  |  |  | |  |  |  |  |  |  |  |  |  |  |  |
| 104 |  |  |  | 1 |  |  |  |  |  |  |  |  |  |  |  |  |  | |  |  |  |  |  |  |  |  |  |  |  |
| 105 |  |  |  | 1 |  |  |  |  |  |  |  |  |  |  |  |  |  | |  |  |  |  |  |  |  |  |  |  |  |
| 106 |  |  |  | 1 |  |  |  |  |  |  |  |  |  |  |  |  |  | |  |  |  |  |  |  |  |  |  |  |  |
| 107 |  |  |  | 1 |  |  |  |  |  |  |  |  |  |  |  |  |  | |  |  |  |  |  |  |  |  |  |  |  |
| 108 |  |  |  | 1 |  |  |  |  |  |  |  |  |  |  |  |  |  | |  |  |  |  |  |  |  |  |  |  |  |
| 109 |  |  |  |  |  |  | 1 |  |  | 1 |  |  |  |  |  |  |  | |  |  |  |  |  |  |  |  |  |  |  |
| 110 |  |  |  |  |  |  |  |  |  | 1 |  |  |  |  |  |  |  | |  |  |  |  |  |  |  |  |  |  |  |
| 111 |  |  |  |  |  |  |  |  |  | 1 |  |  |  |  |  |  |  | |  |  |  |  |  |  |  |  |  |  |  |
| 112 |  |  |  |  |  |  |  |  |  |  |  |  |  |  |  |  |  | |  |  |  |  |  | 1 |  |  |  |  |  |
| 113 |  |  |  |  |  |  |  |  |  |  |  |  |  |  |  |  |  | |  |  |  |  |  | 1 |  |  |  |  |  |
| 114 |  |  |  |  |  |  | 1 |  |  |  |  |  |  |  |  |  |  | |  |  |  |  |  | 1 |  |  |  |  |  |
| 115 |  |  |  |  |  |  |  |  |  |  |  |  |  |  |  |  |  | |  |  |  |  |  | 1 |  |  |  |  |  |
| 116 |  |  |  |  |  |  |  |  |  |  |  |  |  |  |  |  |  | |  |  |  |  |  | 1 |  |  |  |  |  |
| 117 |  |  |  |  | 1 |  |  |  |  |  |  |  |  |  |  |  |  | |  |  |  |  |  |  |  |  |  |  |  |
| 118 |  |  |  |  | 1 |  |  |  |  |  |  |  |  |  |  |  |  | |  |  |  |  |  |  |  |  |  |  |  |
| 119 |  |  |  |  |  |  |  |  |  |  |  |  |  |  |  |  |  | |  |  | 1 |  |  |  |  |  |  |  |  |
| 120 |  |  |  |  |  |  |  |  |  |  |  |  |  |  |  |  |  | |  |  | 1 |  |  |  |  |  |  |  |  |
| 121 |  |  |  |  |  |  |  |  |  |  |  |  |  |  |  |  |  | |  |  | 1 |  |  |  |  |  |  |  |  |
| 122 |  |  |  |  |  |  |  |  |  |  |  |  |  |  | 1 |  |  | |  |  |  |  |  |  |  |  |  |  |  |
| 123 |  |  |  |  |  |  |  |  |  |  |  |  |  |  | 2 |  |  | |  |  |  |  |  |  |  |  |  |  |  |
| 124 |  |  |  |  |  |  |  |  |  |  |  |  |  |  | 1 |  |  | |  |  |  |  |  |  |  |  |  |  |  |
| 125 |  |  |  |  |  |  |  |  |  |  |  |  |  |  | 1 |  |  | |  |  |  |  |  |  |  |  |  |  |  |
| 126 |  |  |  |  |  |  |  |  |  |  |  |  |  |  | 2 |  |  | |  |  |  |  |  |  |  |  |  |  |  |
| 127 |  |  |  |  |  |  |  |  |  |  |  |  |  |  | 1 |  |  | |  |  |  |  |  |  |  |  |  |  |  |
| 128 |  |  |  |  |  |  |  |  |  |  |  |  |  |  | 1 |  |  | |  |  |  |  |  |  |  |  |  |  |  |
| 129 |  |  |  |  |  |  |  |  |  |  |  |  |  |  | 1 |  |  | |  |  |  |  |  |  |  |  |  |  |  |
| 130 |  |  |  |  |  |  |  |  |  |  |  |  |  |  | 1 |  |  | |  |  |  |  |  |  |  |  |  |  |  |
| 131 |  |  |  |  |  |  |  |  |  |  |  |  |  |  | 1 |  |  | |  |  |  |  |  |  |  |  |  |  |  |
| 132 |  |  |  |  |  |  |  |  |  |  |  |  |  |  | 1 |  |  | |  |  |  |  |  |  |  |  |  |  |  |
| 133 |  |  |  |  |  | 1 |  |  |  |  |  |  |  |  |  |  |  | |  |  |  |  |  |  |  |  |  |  |  |
| 134 |  |  |  |  |  | 1 |  |  |  |  |  |  |  |  |  |  |  | |  |  |  |  |  |  |  |  |  |  |  |
| 135 |  |  |  |  |  | 1 |  |  |  |  |  |  |  |  |  |  |  | |  |  |  |  |  |  |  |  |  |  |  |
| 136 |  |  |  |  |  | 1 |  |  |  |  |  |  |  |  |  |  |  | |  |  |  |  |  |  |  |  |  |  |  |
| 137 |  | 1 |  |  |  |  |  |  |  |  |  |  |  |  |  |  |  | |  |  |  |  |  |  |  |  |  |  |  |
| 138 |  | 1 |  |  |  |  |  |  |  |  |  |  |  |  |  |  |  | |  |  |  |  |  |  |  |  |  |  |  |
| 139 |  | 1 |  |  |  |  |  |  |  |  |  |  |  |  |  |  |  | |  |  |  |  |  |  |  |  |  |  |  |
| 140 |  | 1 |  |  |  |  |  |  |  |  |  |  |  |  |  |  |  | |  |  |  |  |  |  |  |  |  |  |  |
| 141 |  |  |  |  |  |  |  |  |  |  |  |  |  |  |  |  |  | |  |  |  |  |  |  |  |  |  | 1 |  |
| 142 |  |  |  |  |  |  |  |  |  |  |  |  |  |  |  |  |  | |  | 1 |  |  |  |  |  |  |  |  |  |
| 143 |  |  |  |  |  |  |  |  |  |  |  |  |  |  |  |  |  | |  | 1 |  |  |  |  |  |  |  |  |  |
| 144 |  |  |  |  |  |  |  |  |  |  |  |  |  |  |  |  |  | |  | 1 |  |  |  |  |  |  |  |  |  |
| 145 |  |  |  |  |  |  |  |  |  |  |  |  |  |  |  |  |  | |  | 1 |  |  |  |  |  |  |  |  |  |
| 146 |  |  |  |  |  |  |  |  |  |  |  |  |  |  |  |  |  | |  |  |  |  |  |  |  | 1 |  |  |  |
| 147 |  |  |  |  |  |  |  |  |  |  |  |  |  |  |  |  |  | |  |  |  |  |  |  |  | 1 |  |  |  |
| 148 |  |  |  |  |  |  |  |  |  |  |  |  |  |  |  |  |  | |  |  |  |  |  |  |  | 1 |  |  |  |
| 149 |  |  |  |  |  |  |  |  |  |  |  |  |  |  |  |  |  | |  |  |  |  |  |  |  | 1 |  |  |  |
| 150 |  |  |  |  |  |  |  |  |  |  |  |  |  |  |  |  |  | |  |  |  |  |  |  |  | 1 |  |  |  |
| 151 |  |  |  |  |  |  |  |  |  |  | 1 |  |  |  |  |  |  | |  |  |  |  |  |  |  |  |  |  |  |
| 152 |  |  |  |  |  |  |  |  |  |  | 1 |  |  |  |  |  |  | |  |  |  |  |  |  |  |  |  |  |  |
| 153 |  |  |  |  |  |  |  |  |  |  |  | 1 |  |  |  |  |  | |  |  |  |  |  |  |  |  |  |  |  |
| 154 |  |  |  |  |  |  |  |  |  |  |  | 1 |  |  |  |  |  | |  |  |  |  |  |  |  |  |  |  |  |
| 155 |  |  |  |  |  |  |  |  |  |  |  | 1 |  |  |  |  |  | |  |  |  |  |  |  |  |  |  |  |  |
| 156 |  |  |  |  |  |  | 1 |  |  |  |  |  |  |  |  |  |  | |  |  |  |  |  |  |  |  |  |  |  |
| 157 |  |  |  |  |  |  | 1 |  |  |  |  |  |  |  |  |  |  | |  |  |  |  |  |  |  |  |  |  |  |
| 158 |  |  |  |  |  |  |  |  |  |  |  |  | 1 |  |  |  |  | |  |  |  |  |  |  |  |  |  |  |  |
| 159 |  |  |  |  |  |  |  |  |  |  |  |  | 1 |  |  |  |  | |  |  |  |  |  |  |  |  |  |  |  |
| 160 |  |  |  |  |  |  |  |  |  |  |  |  | 1 |  |  |  |  | |  |  |  |  |  |  |  |  |  |  |  |
| 161 |  |  |  |  |  |  |  |  |  |  |  |  | 1 |  |  |  |  | |  |  |  |  |  |  |  |  |  |  |  |
| 162 |  |  |  |  |  |  |  |  |  |  |  |  | 1 |  |  |  |  | |  |  |  |  |  |  |  |  |  |  |  |
| 163 |  |  |  |  |  |  |  |  |  |  |  |  | 1 |  |  |  |  | |  |  |  |  |  |  |  |  |  |  |  |
| 164 |  |  |  |  |  |  |  |  |  |  |  |  | 1 |  |  |  |  | |  |  |  |  |  |  |  |  |  |  |  |
| 165 |  |  |  |  |  |  |  |  |  |  |  |  | 1 |  |  |  |  | |  |  |  |  |  |  |  |  |  |  |  |
| TOTALS | 15 | 22 | 17 | 21 | 17 | 15 | 18 | 15 | 14 | 17 | 13 | 18 | 17 | 15 | 16 | 18 | 19 | | 5 | 14 | 14 | 15 | 16 | 24 | 20 | 24 | 18 | 4 |  |

**APPENDIX C: *Tritia obsoleta* 18S hapolotype occurrences and site totals in the native east coast and introduced west coast.** See above for sites and subregions.

| **HAP** | **North American East Coast** | | | | | | | | | | | | | | | **North American West Coast** | | | | | | | |
| --- | --- | --- | --- | --- | --- | --- | --- | --- | --- | --- | --- | --- | --- | --- | --- | --- | --- | --- | --- | --- | --- | --- | --- |
|  | **HAR** | **POR** | **IP** | **JEL** | **MYS** | **PJ** | **WR** | **GS** | **SHIN** | **LEW** | **VB** | **WACH** | **WNC** | **AB** | **GA** | **BLS** | **SBD** | **101** | **COY** | **MLK** | **SL** | **HAY** | **RWC** |
| 1 | 1 | 2 |  | 3 | 2 | 2 | 2 | 2 | 1 | 3 | 5 | 4 | 2 | 1 | 5 | 2 | 5 | 8 | 2 | 1 | 4 | 4 | 2 |
| 2 |  |  |  |  |  |  |  |  |  |  |  |  |  |  |  |  |  |  | 1 |  |  |  |  |
| 3 |  |  | 1 | 1 |  |  |  |  |  |  |  |  |  |  |  |  |  |  |  |  |  |  |  |
| 4 |  |  |  |  |  | 1 |  |  |  |  |  |  |  |  |  |  |  |  |  | 1 |  |  |  |
| 5 |  |  |  |  |  |  |  |  |  |  | 1 |  |  |  |  |  |  |  |  |  |  |  |  |
| SUM | 1 | 2 | 1 | 4 | 2 | 3 | 2 | 2 | 1 | 3 | 6 | 4 | 2 | 1 | 5 | 2 | 5 | 8 | 3 | 2 | 4 | 4 | 2 |

**APPENDIX D: Trematode COI hapolotype occurrences and site totals in the native east coast and introduced west coast for the four trematode species included in the study (*Austrobilharzia variglandis* (*AV*)*, Himasthla quissitensis* (*HQ*)*, Lepocreadium setiferoides (LS)*, and *Zoogonus lasius* (*ZL*)).** See above for sites and subregions.

| **Hap** | **North American East Coast** | | | | | | | | | | | | | | **North American West Coast** | | | | | | | |
| --- | --- | --- | --- | --- | --- | --- | --- | --- | --- | --- | --- | --- | --- | --- | --- | --- | --- | --- | --- | --- | --- | --- |
|  | **HAR** | **POR** | **JEL** | **IP** | **MYS** | **WR** | **GS** | **SHIN** | **PJ** | **LEW** | **VB** | **WACH** | **WNC** | **GA** | **BLS** | **EHP** | **SBD** | **NAH** | **HAY** | **SL** | **MLK** | **BFI** |
| AV 1 |  |  |  |  |  |  |  |  |  | 3 |  |  |  |  | 5 |  |  | 1 |  |  |  |  |
| AV 2 |  | 1 |  |  |  |  |  |  |  | 1 |  | 1 |  |  | 8 | 1 |  |  | 2 |  |  |  |
| AV 3 |  |  |  |  |  |  |  |  |  |  |  |  |  |  | 1 |  |  |  |  |  |  |  |
| AV 4 |  |  |  |  |  |  |  |  |  | 1 |  |  |  |  |  |  |  |  |  |  |  |  |
| AV 5 |  |  |  |  |  |  |  |  |  |  |  |  |  |  |  |  |  | 1 |  |  |  |  |
| AV 6 |  | 1 |  |  |  |  |  |  |  |  |  |  |  |  |  |  |  |  |  |  |  |  |
| AV 7 |  | 1 |  |  |  |  |  |  |  |  |  |  |  |  |  |  |  |  |  |  |  |  |
| AV 8 |  | 1 |  |  |  |  |  |  |  |  |  |  |  |  |  |  |  |  |  |  |  |  |
| AV 9 |  | 1 |  |  |  |  |  |  |  |  |  |  |  |  |  |  |  |  |  |  |  |  |
| HQ 1 |  | 1 |  |  |  |  |  | 1 |  |  |  |  |  |  |  |  |  |  | 1 |  |  |  |
| HQ 2 |  |  |  |  |  |  |  | 1 |  |  |  |  |  |  |  |  |  |  |  |  |  |  |
| HQ 3 |  |  |  |  |  |  |  | 2 |  |  |  |  |  |  |  |  |  |  | 3 |  |  |  |
| HQ 4 |  |  |  |  |  |  |  |  |  |  |  |  |  |  |  |  |  |  | 13 | 2 |  |  |
| HQ 5 |  |  |  |  |  |  |  |  |  |  |  |  |  |  |  |  |  |  | 1 |  |  |  |
| HQ 6 |  | 1 |  |  |  |  |  |  |  |  |  |  |  |  |  |  |  |  |  |  |  |  |
| HQ 7 |  |  |  |  |  |  |  |  |  |  |  |  |  |  |  |  |  |  | 1 |  |  |  |
| HQ 8 | 1 |  |  |  |  |  |  |  |  |  |  |  |  |  |  |  |  |  |  |  |  |  |
| HQ 9 |  |  |  |  |  |  | 1 |  |  |  |  |  |  |  |  |  |  |  |  |  |  |  |
| HQ 10 |  | 2 |  |  |  |  |  | 2 |  |  |  |  |  |  |  |  |  |  | 3 | 1 |  |  |
| HQ 11 | 1 |  |  |  |  |  |  |  |  |  |  |  |  |  |  |  |  |  |  |  |  |  |
| HQ 12 |  |  |  |  |  |  |  | 1 |  |  |  |  |  |  |  |  |  |  |  |  |  |  |
| HQ 13 |  |  |  |  |  |  |  | 1 |  |  |  |  |  |  |  |  |  |  |  |  |  |  |
| HQ 14 |  |  |  |  |  |  | 1 |  |  |  |  |  |  |  |  |  |  |  |  |  |  |  |
| HQ 15 |  | 3 |  |  |  |  | 2 |  |  |  |  |  |  |  |  |  |  |  | 3 | 3 |  |  |
| HQ 16 |  |  |  |  |  |  | 1 |  |  |  |  |  |  |  |  |  |  |  |  |  |  |  |
| HQ 17 |  |  | 1 |  |  |  |  |  |  |  |  |  |  |  |  |  |  |  |  |  |  |  |
| HQ 18 |  | 1 |  |  |  |  |  |  |  |  |  |  |  |  |  |  |  |  |  |  |  |  |
| HQ 19 | 2 |  |  |  |  |  |  |  |  |  |  |  |  |  |  |  |  |  |  |  |  |  |
| HQ 20 |  |  |  |  |  |  | 2 |  |  |  |  |  |  |  |  |  |  |  |  |  |  |  |
| HQ 21 | 1 |  |  |  |  | 1 |  |  |  |  |  |  |  |  | 1 |  |  |  |  |  |  |  |
| HQ 22 | 1 |  |  |  |  |  |  |  |  |  |  |  |  |  |  |  |  |  |  |  |  |  |
| HQ 23 |  |  |  |  |  |  |  |  |  |  |  |  | 1 |  |  |  |  |  |  |  |  |  |
| HQ 24 |  | 1 |  |  |  |  |  |  |  |  |  |  |  |  | 2 |  |  |  |  |  |  |  |
| HQ 25 |  | 2 |  |  |  |  |  |  |  |  |  |  |  |  |  |  |  |  | 1 |  |  |  |
| HQ 26 |  | 1 |  |  |  |  |  |  |  |  |  |  |  |  |  |  |  |  |  |  |  |  |
| HQ 27 |  |  |  |  |  | 1 |  |  |  |  |  |  |  |  |  |  |  |  |  |  |  |  |
| HQ 28 |  |  |  |  |  | 1 |  |  |  |  |  |  |  |  |  |  |  |  |  |  |  |  |
| HQ 29 | 1 | 1 | 1 |  |  | 2 | 4 | 1 |  |  |  |  |  |  |  |  |  |  | 4 | 1 |  |  |
| HQ 30 |  |  |  |  |  |  |  | 1 |  |  |  |  |  |  |  |  |  |  |  |  |  |  |
| HQ 31 |  |  |  |  |  |  | 1 |  |  |  |  |  |  |  |  |  |  |  |  |  |  |  |
| HQ 32 | 1 |  |  |  |  |  |  |  |  |  |  |  |  |  |  |  |  |  |  |  |  |  |
| HQ 33 |  |  |  |  |  | 1 |  |  |  |  |  |  |  |  |  |  |  |  |  |  |  |  |
| HQ 34 |  |  |  |  |  |  |  |  |  |  |  |  |  |  |  |  |  |  | 2 |  |  |  |
| HQ 35 |  |  |  | 1 |  |  |  |  |  |  |  |  |  |  |  |  |  |  |  |  |  |  |
| HQ 36 |  | 1 |  |  |  |  |  |  |  |  |  |  |  |  |  |  |  |  |  |  |  |  |
| LS1 |  |  |  |  |  |  |  |  | 1 |  |  |  |  |  |  |  |  |  |  |  |  |  |
| LS2 | 1 |  | 4 |  | 2 | 3 | 1 | 1 | 1 |  | 1 |  | 2 |  |  |  |  |  |  |  |  |  |
| LS3 |  |  |  |  | 2 | 1 |  |  |  |  |  |  |  |  |  |  |  |  |  |  |  |  |
| LS4 |  |  |  |  |  | 1 |  |  |  |  |  |  |  |  |  |  |  |  |  |  |  |  |
| LS5 |  |  |  |  |  | 1 |  |  |  |  |  |  |  |  |  |  |  |  |  |  |  |  |
| LS6 |  |  |  |  |  |  | 1 |  |  |  |  |  |  |  |  |  |  |  |  |  |  |  |
| LS7 |  |  |  |  | 1 |  |  |  |  |  |  |  |  |  |  |  |  |  |  |  |  |  |
| LS8 |  |  |  |  |  |  |  |  |  |  | 2 |  |  |  |  |  |  |  |  |  |  |  |
| LS9 |  |  |  |  |  |  |  |  |  |  | 1 |  |  |  |  |  |  |  |  |  |  |  |
| LS10 |  |  |  |  | 1 |  |  |  |  |  |  |  |  |  |  |  |  |  |  |  |  |  |
| LS11 |  |  |  |  |  | 1 |  |  |  |  |  |  |  |  |  |  |  |  |  |  |  |  |
| LS12 |  |  |  |  |  |  |  |  |  | 1 |  |  |  |  |  |  |  |  |  |  |  |  |
| LS13 |  |  |  |  | 1 |  |  |  |  |  |  |  |  |  |  |  |  |  |  |  |  |  |
| LS14 |  |  |  |  |  |  |  |  |  |  |  |  |  | 1 |  |  |  |  |  |  |  |  |
| LS15 |  |  |  |  |  |  |  |  | 1 |  |  |  |  |  |  |  |  |  |  |  |  |  |
| LS16 |  |  |  |  | 1 |  |  |  |  |  |  |  |  |  |  |  |  |  |  |  |  |  |
| LS17 |  |  |  |  |  |  | 1 |  |  |  |  |  |  |  |  |  |  |  |  |  |  |  |
| LS18 |  |  |  |  |  | 1 |  |  |  |  |  |  |  |  |  |  |  |  |  |  |  |  |
| LS19 |  |  |  |  |  | 1 |  |  |  |  |  |  |  |  |  |  |  |  |  |  |  |  |
| LS20 |  |  |  |  |  | 2 |  |  |  |  |  |  |  |  |  |  |  |  |  |  |  |  |
| LS21 |  |  |  |  |  |  |  |  |  |  | 1 |  |  |  |  |  |  |  |  |  |  |  |
| LS22 |  |  |  |  |  |  |  |  |  |  |  |  |  |  |  |  |  |  |  |  | 1 |  |
| LS23 |  |  |  |  |  |  | 1 |  |  |  |  |  |  |  |  |  |  |  |  |  |  |  |
| LS24 |  |  |  |  |  |  |  |  |  |  |  |  |  |  |  |  |  |  |  |  |  | 1 |
| LS25 |  |  |  |  |  |  |  |  |  | 1 |  |  | 1 |  |  |  |  |  |  |  |  |  |
| LS26 |  |  |  |  |  |  |  |  |  |  |  |  | 1 |  |  |  |  |  |  |  |  |  |
| LS27 |  |  |  |  |  |  |  |  |  |  |  |  |  |  |  |  |  |  | 1 |  |  |  |
| LS28 |  |  |  |  |  |  |  |  |  | 1 |  |  |  |  |  |  |  |  |  |  |  |  |
| LS29 |  |  |  |  |  |  |  |  |  | 1 |  |  | 6 |  |  |  |  |  |  |  |  |  |
| LS30 |  |  |  |  |  |  |  |  |  |  | 1 |  |  |  |  |  |  |  |  |  |  |  |
| LS31 | 1 |  |  |  |  |  |  |  |  |  |  |  |  |  |  |  |  |  |  |  |  |  |
| LS32 |  |  |  |  |  |  |  |  |  |  | 1 |  |  |  |  |  |  |  |  |  |  |  |
| LS33 |  |  |  |  |  |  |  |  |  |  | 1 |  |  |  |  |  |  |  |  |  |  |  |
| LS34 |  |  |  |  |  |  |  |  |  |  | 1 |  |  |  |  |  |  |  |  |  |  |  |
| LS35 |  | 1 |  |  |  |  |  |  |  |  |  |  |  |  |  |  |  |  |  |  |  |  |
| LS36 |  |  |  |  |  |  |  |  |  |  | 1 |  | 1 |  |  |  |  |  |  |  |  |  |
| LS37 |  |  |  |  |  |  |  |  |  | 1 |  |  |  |  |  |  |  |  |  |  |  |  |
| LS38 |  |  |  |  |  |  |  |  |  |  |  |  |  |  |  |  |  |  |  | 1 |  |  |
| LS39 |  |  |  |  |  |  |  |  |  |  |  |  |  |  |  |  |  |  |  | 1 |  |  |
| LS40 |  |  |  |  |  |  |  |  | 1 |  |  |  |  |  |  |  |  |  |  |  |  |  |
| LS41 |  |  |  |  |  |  |  |  |  |  |  |  |  |  |  |  |  |  | 1 |  |  |  |
| LS42 |  |  |  |  |  |  |  |  |  |  |  |  |  |  |  |  |  |  |  | 1 |  |  |
| LS43 |  |  |  |  |  |  |  |  |  |  | 1 |  |  |  |  |  |  |  |  |  |  |  |
| LS44 |  |  |  |  |  |  |  |  |  |  |  |  | 1 |  |  |  |  |  |  |  |  |  |
| LS45 |  |  |  |  |  |  |  |  |  |  |  |  |  |  |  |  |  |  |  | 1 |  |  |
| ZL1 |  |  | 1 |  |  |  |  |  |  |  |  |  |  |  |  |  |  |  |  |  |  |  |
| ZL2 |  |  |  |  |  |  |  |  |  | 2 |  |  |  |  |  |  |  |  |  |  |  |  |
| ZL3 |  |  |  |  |  |  |  |  |  | 1 |  |  |  |  |  |  |  |  |  |  |  |  |
| ZL4 |  |  |  |  |  |  |  |  |  | 1 |  |  |  |  |  |  |  |  |  |  |  |  |
| ZL5 |  |  |  |  |  |  | 1 |  |  |  |  |  |  |  |  |  |  |  |  |  |  |  |
| ZL6 |  |  |  |  | 2 | 1 | 1 | 3 |  |  | 2 |  |  |  |  |  |  |  |  |  |  |  |
| ZL7 |  |  |  |  |  |  |  |  |  |  |  |  |  |  |  |  |  |  | 1 |  |  |  |
| ZL8 |  |  |  |  |  |  |  |  |  | 1 |  |  |  |  |  |  |  |  |  |  |  |  |
| ZL9 |  |  |  |  |  |  |  |  |  |  | 1 |  |  |  |  |  |  |  |  |  |  |  |
| ZL10 |  |  |  |  |  |  |  |  |  |  | 1 |  |  |  |  |  |  |  |  |  |  |  |
| ZL11 |  |  |  |  |  |  |  |  |  |  | 1 |  | 1 |  |  |  |  |  |  |  |  |  |
| ZL12 |  |  |  |  |  |  |  |  |  | 1 |  |  |  |  |  |  |  |  |  |  |  |  |
| ZL13 |  |  |  |  |  |  |  |  |  | 1 |  |  |  |  |  |  |  |  |  |  |  |  |
| ZL14 |  | 1 |  |  |  |  |  |  |  |  | 1 |  |  |  |  |  |  |  |  |  |  |  |
| ZL15 |  |  |  |  |  |  |  |  |  | 1 |  |  |  |  |  |  |  |  |  |  |  |  |
| ZL16 |  |  |  |  |  |  |  |  |  | 1 |  |  |  |  |  |  |  |  |  |  |  |  |
| ZL17 |  |  |  |  |  |  |  |  |  | 1 |  |  |  |  |  |  |  |  |  | 1 |  |  |
| ZL18 |  |  |  |  |  |  |  |  |  |  | 1 |  | 1 |  |  |  |  |  |  |  |  |  |
| ZL19 |  |  |  |  |  |  |  |  |  | 1 |  |  |  |  |  |  |  |  |  |  |  |  |
| ZL20 |  |  |  |  |  |  |  |  |  | 1 |  |  |  |  |  |  |  |  |  |  |  |  |
| ZL21 |  |  |  |  |  |  | 1 |  |  |  |  |  |  |  |  |  |  |  |  |  |  |  |
| ZL22 |  | 3 |  |  | 1 | 2 | 4 |  |  | 17 | 5 | 2 | 7 | 2 |  |  |  | 1 | 2 | 2 | 2 |  |
| ZL23 |  |  |  |  | 1 |  |  |  |  | 1 |  |  |  |  |  |  |  |  |  |  |  |  |
| ZL24 |  |  |  |  |  |  |  |  |  |  | 1 | 1 |  |  |  |  |  |  |  |  |  |  |
| ZL25 |  |  |  |  |  |  |  |  |  |  | 1 |  |  |  |  |  |  |  |  |  |  |  |
| ZL26 |  |  |  |  |  |  |  |  |  |  |  |  | 1 |  |  |  |  |  |  |  |  |  |
| ZL27 |  |  |  |  |  |  |  |  |  |  |  |  | 1 |  |  |  |  |  |  |  |  |  |
| ZL28 |  |  |  |  |  |  |  |  |  |  | 1 |  |  |  |  |  |  |  |  |  |  |  |
| ZL29 | 1 |  |  |  |  |  |  |  |  |  |  |  |  |  |  |  |  |  |  | 1 |  |  |
| ZL30 |  |  |  |  |  |  |  |  |  | 1 |  |  |  |  |  |  |  |  |  |  |  |  |
| ZL31 |  |  |  |  |  |  |  |  |  |  |  |  | 1 |  |  |  |  |  |  |  |  |  |
| ZL32 |  |  |  |  |  |  |  |  |  | 2 |  |  |  |  |  |  |  |  |  |  |  |  |
| ZL33 |  |  |  |  |  |  |  |  |  |  |  | 1 |  |  |  |  |  |  |  |  |  |  |
| ZL34 |  |  |  |  |  |  |  |  |  |  |  |  | 1 |  |  |  |  |  |  |  |  |  |
| ZL35 |  |  |  |  | 1 |  |  |  |  | 6 | 1 | 1 |  |  |  |  |  |  |  |  |  |  |
| ZL36 |  |  |  |  |  |  |  |  |  | 1 |  |  |  |  |  |  |  |  |  |  |  |  |
| ZL37 |  |  |  |  |  |  |  |  |  |  |  |  | 1 |  |  |  |  |  |  |  |  |  |
| ZL38 |  |  |  |  |  |  |  |  |  | 1 |  |  |  |  |  |  |  |  |  |  |  |  |
| ZL39 |  |  |  |  |  |  |  |  |  |  | 1 |  |  |  |  |  |  |  |  |  |  |  |
| ZL40 |  |  |  |  |  |  |  |  |  |  |  |  | 1 |  |  |  |  |  |  |  |  |  |
| ZL41 |  |  |  |  |  |  |  |  |  |  |  |  | 1 |  |  |  |  |  |  |  |  |  |
| ZL42 |  |  |  |  |  |  |  |  |  | 1 |  |  |  |  |  |  |  |  |  |  |  |  |
| ZL43 |  |  |  |  |  |  |  |  |  | 1 |  |  |  |  |  |  |  |  |  |  |  |  |
| ZL44 |  |  |  |  |  |  |  |  |  | 1 |  |  |  |  |  |  |  |  |  |  |  |  |
| ZL45 |  |  |  |  |  |  |  |  |  | 1 |  |  |  |  |  |  |  |  |  |  |  |  |
| TOTALS | 11 | 24 | 7 | 1 | 13 | 20 | 23 | 14 | 4 | 55 | 28 | 6 | 29 | 3 | 17 | 1 | 0 | 3 | 39 | 15 | 3 | 1 |

**APPENDIX E: *Himasthla quissitensis* (*HQ*) 18S** **hapolotype occurrences and site totals in the native east coast and introduced west coast for *HQ***. See above for sites and subregions.

| **HAP** | **North American East Coast** | | | | | **North American West Coast** | | |
| --- | --- | --- | --- | --- | --- | --- | --- | --- |
|  | **HAR** | **POR** | **WR** | **GS** | **SHIN** | **BLS** | **HAY** | **SL** |
| 1 |  | 3 | 2 | 2 |  | 2 | 2 | 3 |
| 2 |  |  |  | 1 |  |  |  |  |
| 3 |  |  |  | 2 |  |  |  |  |
| 4 |  |  |  | 1 |  |  |  |  |
| 5 | 1 |  |  |  |  |  |  |  |
| 6 |  |  |  |  |  |  | 1 | 2 |
| 7 |  |  |  |  |  |  | 1 |  |
| 8 |  |  |  |  |  |  | 1 |  |
| 9 |  |  |  |  | 1 |  |  |  |
| 10 |  |  | 1 |  |  |  |  |  |
| TOTALS | 1 | 3 | 3 | 6 | 1 | 2 | 5 | 5 |
